# Designing for implementation: development, delivery, and multi-method evaluation of a clinician training program for a novel technology-based concussion assessment tool

**DOI:** 10.3389/frhs.2026.1937910

**Published:** 2026-10-01

**Authors:** Joshua Shore, Christine Provvidenza, Andrew Lovell, Brintha Ingaramoorthy, Elise Christopoulos, Andrea Hickling, Michael Hutchison, Sarah E. P. Munce, Kathryn J. Schneider, Roger Zemek, Isabelle Gagnon, Elaine Biddiss, Shannon E. Scratch

**Affiliations:** 1 Bloorview Research Institute, Holland Bloorview Kids Rehabilitation Hospital, Toronto, ON, Canada; 2 Rehabilitation Sciences Institute, Temerty Faculty of Medicine, University of Toronto, Toronto, ON, Canada; 3 Holland Bloorview Kids Rehabilitation Hospital, Toronto, ON, Canada; 4 Faculty of Kinesiology and Physical Education, University of Toronto, Toronto, ON, Canada; 5 Department of Occupational Science and Occupational Therapy, University of Toronto, Toronto, ON, Canada; 6 Keenan Research Centre for Biomedical Science, St. Michael's Hospital, Toronto, ON, Canada; 7 Institute of Health Policy, Management, & Evaluation, University of Toronto, Toronto, ON, Canada; 8 Sport Injury Prevention Research Centre, Faculty of Kinesiology, University of Calgary, Calgary, AB, Canada; 9 Alberta Children's Hospital Research Institute, University of Calgary, Calgary, AB, Canada; 10 Hotchkiss Brain Institute, University of Calgary, Calgary, AB, Canada; 11 Department of Pediatrics and Emergency Medicine, University of Ottawa, Ottawa, ON, Canada; 12 Children's Hospital of Eastern Ontario, Ottawa, ON, Canada; 13 School of Physical and Occupational Therapy, McGill University, Montreal, QC, Canada; 14 Department of Pediatrics, McGill University Health Centre, Montreal, QC, Canada; 15 Institute of Biomedical Engineering, Temerty Faculty of Medicine, University of Toronto, Toronto, ON, Canada; 16 Department of Pediatrics, Temerty Faculty of Medicine, University of Toronto, Toronto, ON, Canada

**Keywords:** curriculum development, digital health technology, evaluation study, healthcare education, implementation, needs assessment, rehabilitation, training

## Abstract

**Background:**

Implementation strategies like user training are often excluded from research activities for digital health technologies (DHTs) or reserved for advanced stages of evidence generation. This may contribute to challenges in clinical translation due to usability, feasibility, fidelity, and scalability issues. Using the example of a concussion assessment tool called *R2Play,* we demonstrate how clinicians can be engaged in developing training resources for DHTs during early stages of evidence generation and illustrate potential benefits of this approach.

**Objectives:**

(1) Develop a clinician training program for *R2Play* administration; (2) Deliver the *R2Play* training program to clinician participants across multiple research sites; (3) Evaluate the training program based on participant reactions and learning using a multi-method approach.

**Methods:**

The Kern Model for Medical Curricula guided training development, which involved a needs assessment, specifying learning objectives, and selecting education strategies. Training was delivered to clinician participants at four sites in two parts: (1) asynchronous online learning modules (1 h); and (2) live training session to practice *R2Play* administration tasks (1 h). Evaluation measures based on the Kirkpatrick Model for Training Evaluation included module-embedded knowledge check questions, live training task completion scores, post-training survey, and follow-up interview. Quantitative measures were analyzed descriptively (frequencies, proportions, means), and content analysis was applied to interview data.

**Results:**

Eighteen clinicians participated (*n* = 15 women), primarily physiotherapists (*n* = 11) and athletic therapists (*n* = 3). Survey and interview feedback generally indicated positive reactions (satisfaction, relevance, engagement) and learning perceptions (knowledge, skills, confidence). Participants objectively and subjectively experienced most difficulty in learning system set-up, delivering precise assessment level instructions, and explaining assessment scoring metrics. Interview data highlighted topics requiring additional content (navigating software interface, interpreting results, troubleshooting). Suggested improvements included an end-to-end workflow demonstration, mock run-through, and opportunities to observe experienced users.

**Conclusions:**

Training programs for DHTs should consider addressing technical proficiency (i.e., system set-up and operation), clinical reasoning (i.e., interpreting data), and communication with care recipients. Early multicenter studies involving end-user training can help enhance the usability of DHTs and identify additional strategies to foster implementation. This work adds to limited empirical literature on how to prepare clinicians for using DHTs through evidence-based practical recommendations.

## Introduction

### Background

Digital health technologies (DHTs), defined as products that use information and communication technologies to enhance healthcare delivery (e.g., mobile health applications, wearable devices, telehealth) (1), offer great potential to address challenges in neurorehabilitation. As assessment tools, DHTs can provide more ecologically relevant test paradigms (2), improve scoring accuracy (3), enable real-world monitoring (4), and provide personalized feedback (2, 4). Within treatment interventions, DHTs may enhance patient engagement in therapy (5), facilitate intensive task-oriented therapy (6), support self-management (7), and improve care access (8). However, translating DHTs into clinical practice remains a significant challenge to adoption (9). Implementation factors that influence DHT adoption in neurorehabilitation practice include characteristics of the patient and/or clinician user (e.g., capability, experience, and beliefs), user experience (i.e., usability), intervention content (i.e., benefits to care), access to the DHT, and available support systems (e.g., training and support) (10).

Co-design and end-user engagement are recommended to ensure these implementation factors are considered in DHT development (11, 12). As primary users of DHTs, clinicians play an important role in their adoption and have an interest in their development and evaluation (13). However, user involvement in DHT research is often confined to discrete stages (e.g., usability testing), with historically less participation in other phases (e.g., design and validation), which limits their opportunity to affect change (14). Moreover, clinician perspectives are typically sought in relation to the DHT itself, with less attention to accompanying support components required for real-world use (e.g., user training). Despite their importance for implementation (10), these support components are often ad-hoc additions that are rarely developed or evaluated using systematic methods.

### Clinician training for DHTs

Education and training are the most common implementation strategies to facilitate adoption of DHTs in rehabilitation practice (15). This is understandable since low knowledge and confidence are common barriers to DHT adoption by clinicians (13, 16–18). It also reflects a broader proclivity towards education and training as primary methods for translating evidence-based practices in rehabilitation (19). In implementation contexts, education refers to dissemination of information to increase knowledge about a practice (e.g., awareness of a DHT), while training also includes skill development to acquire competencies necessary to perform the practice (e.g., using a DHT), often using hands-on interactive methods (20). Importantly, training alone is unlikely to produce sustained practice change and additional strategies may be required for successful adoption (19). Nevertheless, training is a crucial component and precursor to other implementation strategies, with meaningful ramifications for the quality and ease with which clinicians can apply DHTs consistently as intended (21). Training is especially important for DHTs that involve equipment set-up, calibration, customization, maintenance, and/or troubleshooting (22).

Methodological guidelines recommend that support services like training and implementation quality (i.e., fidelity) should be considered throughout the course of developing and generating evidence for healthcare innovations like DHTs (22, 23). Yet in practice, implementation is often a post-design activity that is not addressed until after the DHT and/or intervention is firmly established and refined through controlled lab-based studies (12). Accordingly, training resources to prepare clinicians to operate DHTs, and their ability to employ them appropriately, may not be addressed until the tool is evaluated in real-world settings through effectiveness and implementation trials (23). Table 1 outlines the relevance of user training for DHTs during earlier stages of evidence generation, highlighting issues of usability, feasibility, fidelity, and scalability that may contribute to challenges translating DHTs into practice. These conceptual issues were identified based on a critical synthesis of the literature and the authorship team's collective expertise and experience in DHT research and development. Like the DHT itself, effective training may require cycles of design, evaluation, and refinement based on user feedback (24). Thus, purposefully designing for implementation by iteratively creating and testing training resources with clinician users alongside development of DHTs could help facilitate adoption.

**Table 1:** Relevance of user training for digital health technologies (DHTs) and associated interventions during early stages of evidence generation.

Issue	Description
Usability	Usability is specific to the context of use including the extent of training (25). Learnability, a quality of usability, describes how easily users can master system functions and apply this learning to tasks in real-life use (26, 27). Training and usability are closely related; (27, 28) without training, usability cannot be fully understood or optimized, and issues may not be identified until the DHT is more mature, when changes are more difficult and costly. Rapid small-scale testing provides useful insights to enhance learnability and usability of DHTs by improving user training (27, 28). Understanding users' learning needs helps embed support functions within DHTs (e.g., video tutorials, “how to” guides, troubleshooting manuals) (29).
Feasibility	To support generalizability, feasibility should be evaluated with users in realistic contexts that reflect conditions under which the DHT will be used, considering the DHT itself and its delivery processes (e.g., training) (22). Training ensures that users properly operate DHTs, reducing the risk of errors compromising safety during feasibility evaluation (22) When users are trained and feel confident, they are more likely to engage meaningfully with the DHT, (28) which can impact aspects of feasibility (e.g., acceptability, demand, implementation) (30). Trained users provide more contextualized feedback about strengths and weaknesses of the DHT, which is critical for refining the system (22).
Fidelity	Fidelity describes the integrity with which a DHT assessment or intervention is delivered according to its intended protocol (31) and has important implications for validation (22, 32). Standardized training is an integral strategy for maintaining fidelity by ensuring consistent delivery of the DHT (33). In validation and efficacy studies, DHTs are often administered by highly experienced “superusers” who receive resource-intensive training and support, while in effectiveness or implementation trials, training efforts reflect what would typically occur under real-world clinical conditions (34). Without carefully developed training resources, fidelity may be compromised by variability in how DHTs are used in natural settings, threatening the psychometric properties of DHT assessments (35) and/or the validity of efficacy findings for DHT interventions (33). Early focus on training and fidelity allows for iterative re-design of any aspects of the DHT tool that make it difficult to achieve fidelity, and can help inform fidelity monitoring plans in subsequent sutides (36).
Scalability	Efficient processes for developing, evaluating, and scaling DHTs are needed to match the rapid pace of technology advancement (22). If training resources are not adequately refined, scaling the DHT intervention may be delayed, or rollouts may be rushed, leading to poor quality implementation that reduces long-term sustainability. Developing training resources alongside the DHT intervention can help build a comprehensive package for commercialization, enabling rapid deployment once sufficient evidence supports its use in practice. Effective user training lends credibility to the DHT and can improve clinicians' confidence in using it, which may support adoption (37).

### Case example of *R2Play:* A novel technology-based assessment tool for concussion

Using the example of a technology-based concussion assessment tool called *R2Play,* this paper demonstrates how clinicians can be engaged to iteratively design training resources for DHTs during early stages of evidence generation and illustrates potential benefits of this approach for enhancing usability and informing implementation planning.

### Overview of *R2Play* assessment tool

To address a gap in clinically feasible and ecologically relevant assessments of sport concussion recovery in youth (38), our team developed *R2Play,* a technology-based multi-domain assessment tool designed to simulate the demands of sport within clinic settings to inform return-to-play decisions (39). It consists of tablets displaying numbers and letters that participants run between to connect a trail in alphanumerical order (1-A-2-B…6-F) through levels with layered challenges to physical exertion, cognition, and perception (Figure 1)*.* To administer *R2Play*, clinicians use a custom-designed software interface which guides them through tasks including system connection, assessment customization, delivering instructions, documenting observations and client ratings, and reviewing results.

**Figure 1:**
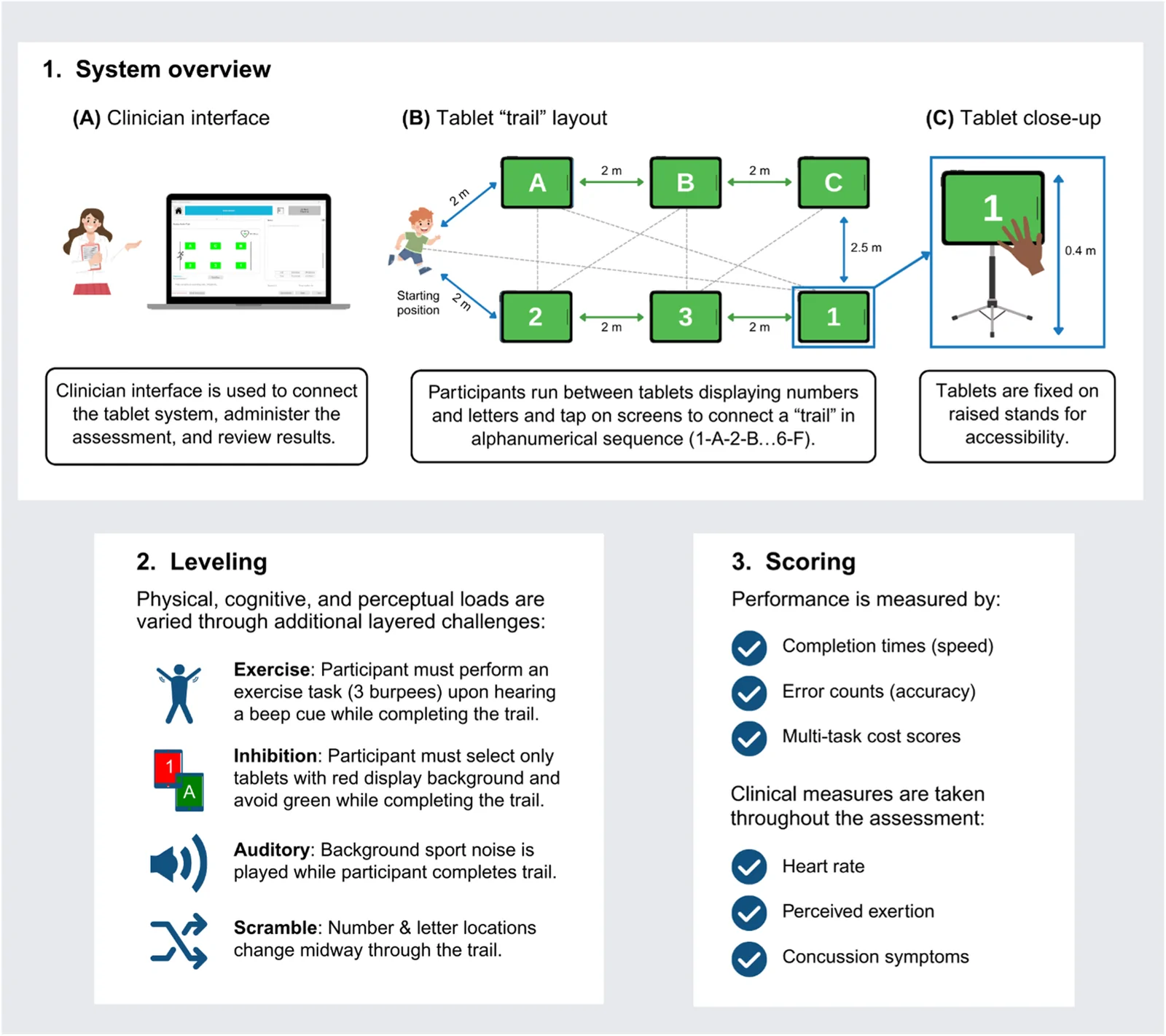
Overview of *R2Play* assessment tool including **(1)** system and assessment concept, **(2)** leveling structure, and **(3)** scoring metrics. Reproduced from Shore et al. (60), Disability and Rehabilitation: Assistive Technology, https://doi.org/10.1080/17483107.2026.2721780, under the terms of the Creative Commons Attribution 4.0 International License (CC BY 4.0).

### Work to date

In preliminary studies using mixed and multi-method designs, we first established proof-of-concept for *R2Play* by demonstrating alignment of the prototype with initial design objectives (e.g., fun for youth, sport-like, clinically valuable) (40), and subsequently confirmed its feasibility at our site, finding excellent usability, vigorous physical exertion demands, and promising qualitative data regarding its potential to fill gaps in clinical practice (41). Importantly, clinicians in these studies received minimal orientation (<60 min) and were closely supported by research team members. Unsurprisingly, clinicians have emphasized the need for training to enable use of *R2Play* in practice (39–41), for example: “*There would have to be training… there would have to be some pretty informative knowledge translation to the clinicians using this.”* [unpublished quotation from previous dataset (39)]. Thus, training resources were needed to enable consistent delivery of *R2Play* in follow-up studies and support future clinical implementation.

In 2024, we launched a multicenter pilot study to evaluate the cross-site feasibility, face validity, and content validity of *R2Play* across five Canadian concussion centers (42). The study involved clinician and youth participants testing *R2Play* at each site, which presented a unique opportunity to begin addressing implementation strategies like user training while *R2Play* was still undergoing iterative testing and refinement. Within the context of the multicenter study, this paper outlines our process of developing and evaluating a clinician training program for *R2Play*.

### Objectives

Guided by the Kern Model for Medical Curricula (43), this study aimed to (1): Develop a training program to prepare clinicians to independently administer a technology-based concussion assessment tool called *R2Play*; (2) Deliver the training program to clinician participants across four research sites; (3) Conduct an initial evaluation of the training program using a multi-method approach based on the Kirkpatrick Model of training evaluation (44).

## Materials and methods

### Study context

This sub-study took place within the context of a larger multicenter project in which *R2Play* is evaluated at five Canadian academic concussion centers (*see Scratch* et al. *for study protocol*) (42). Four sites participated in this sub-study: Holland Bloorview Kids Rehabilitation Hospital in Toronto (lead site), 360 Concussion Care Ottawa, University of Calgary Sport Injury Prevention Research Centre and Sport Medicine Centre, and Montreal Children's Hospital. Clinicians were recruited at each site, trained to deliver *R2Play* as described herein, and paired with two youth participants previously cleared to return-to-play post-concussion to trial *R2Play*. Sites were managed by local research staff who completed the same training program (plus one or more additional independent practice sessions), supported by the lead site. Ethics approvals were secured at each site and participants provided informed written consent.

### Approach

The overarching research approach was guided by the Kern Model for Medical Curricula, six interrelated steps encompassing training creation, delivery, and evaluation (43). We chose the Kern model because of its contextual relevance, alignment with study objectives, and previous use in our institution (45). Figure 2 demonstrates how the Kern Model was applied to *R2Play* training. Activities within each stage are detailed in the subsections below, organized by study objectives.

**Figure 2:**
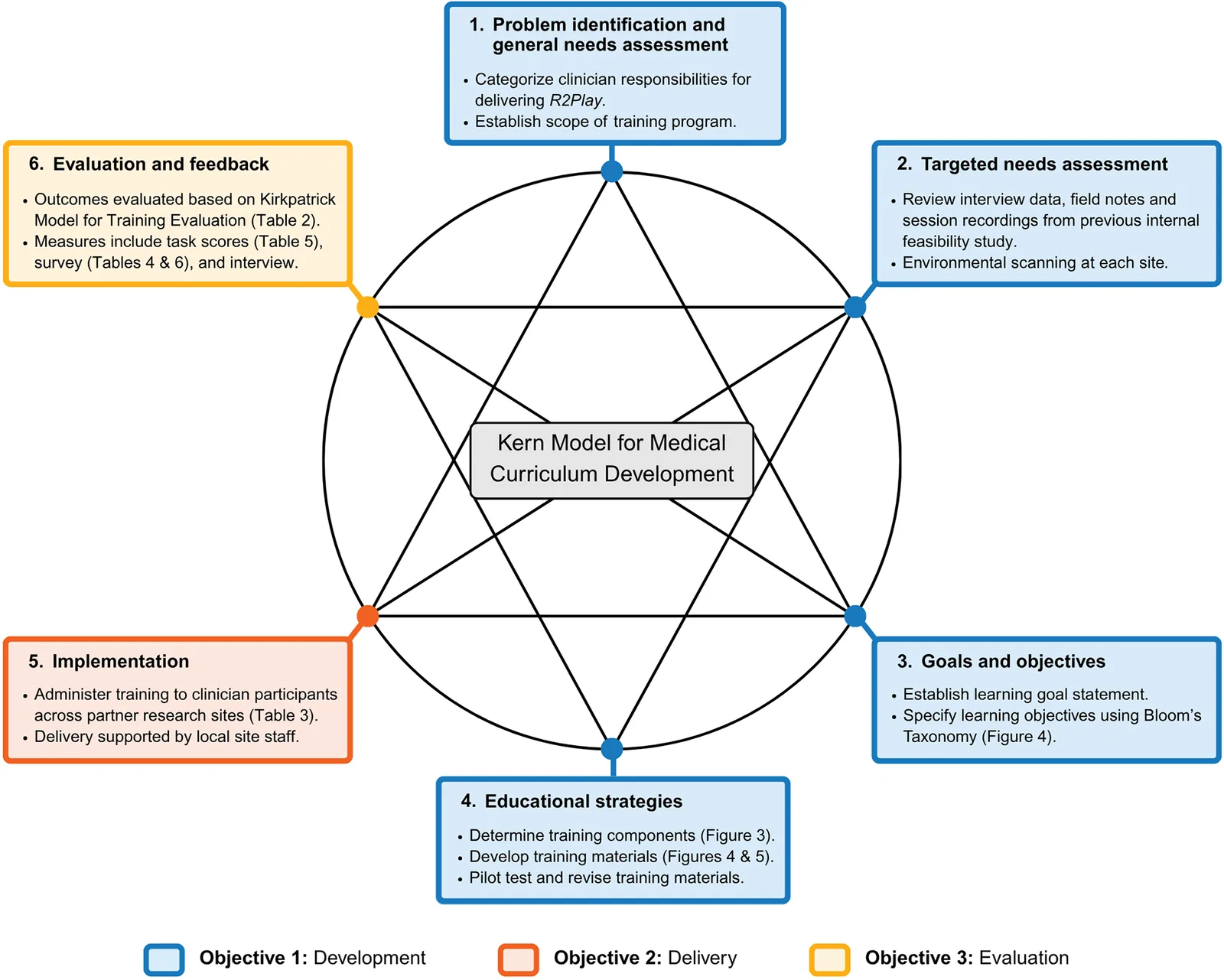
Application of Kern model for medical curricula (43) to *R2Play* training program.

### Objective 1: training development

#### Step 1: problem identification and general needs assessment

The first step was to identify the “problem” (i.e., the need for training) and determine general learning requirements. As outlined above, training was needed to enable clinician participants to deliver *R2Play* at each site. Through team discussion, we categorized responsibilities of clinician participants into three areas for training to address: (1) equipment set-up and system connection; (2) assessment administration; and (3) results interpretation. Given its early stage of development, the clinical significance of *R2Play* scoring is not yet understood, and application in clinical decision making is premature. Therefore, results interpretation in the current training curriculum was limited to conceptual understanding and explanation of scoring metrics.

#### Step 2: targeted needs assessment

Targeted needs assessment involves understanding specific learning needs of the population and the environment in which they learn and practice (43). To assess learning needs, we reviewed data from our previous feasibility study (41) and conducted environmental scans with participating sites.

##### Feasibility study data

In the feasibility study, clinician participants completed a virtual orientation session (1 h) involving a brief didactic presentation, video demonstration of the *R2Play* software interface, and a screen-share activity to practice navigating the interface (41). After two assessment sessions with youth participants, clinicians then completed a follow-up interview that included questions exploring their perceived training needs (i.e., needs they are aware of and can self-identify) (46). Interview findings that informed training development are summarized in Supplementary Table 1.

To provide insight regarding unperceived training needs (needs that learners are unaware of or cannot self-identify) (46), we analyzed field notes and session recordings to identify any usability issues or assistance required by clinicians when administering *R2Play*. Across the orientation activity and assessment sessions, common issues identified were navigating the *R2Play* interface, delivering level instructions, and explaining assessment results, highlighting a need for training to address these aspects. These findings are detailed in the feasibility manuscript (41).

##### Environmental scans

Virtual environmental scans were conducted to understand the unique context of each site for study planning. Members of the lead site (authors AL and JS) met virtually with local site staff to discuss study considerations and observe the space in which *R2Play* would be used. Information was gathered from each site regarding their clinician teams, physical space (size, environment), workflows, and training preferences. These conversations revealed considerable diversity in the disciplines, roles, and experience of clinician learners across sites. Support with system set-up was emphasized, as a permanent space for *R2Play* was not available at each site. One site expressed interest in group training, which was deemed infeasible for the present study.

#### Step 3: learning goals and objectives

Based on the needs assessment, we established the learning goal: *clinician learners can independently set up, deliver, and explain results from an R2Play assessment in a standardized manner without assistance.* Specific learning objectives were formulated using Bloom's Taxonomy of Educational Objectives, a framework for defining learning outcomes by cognitive complexity (47). According to Bloom's Taxonomy, learning outcomes exist on a hierarchy from basic knowledge acquisition (i.e., remembering information) to complex skill development (e.g., analyzing options to make an informed decision). We used Bloom's Taxonomy to inform our learning objective verbiage, which focused on the levels of *knowledge, comprehension, and application*.

#### Step 4: educational strategies

A two-part training program (Figure 3) was developed to address learning objectives through asynchronous modules (1 h) and a live practice session (1 h). Informed by adult learning principles (Andragogy) (48) and implementation science (49), this dynamic format delivers information through multiple means to satisfy diverse learning needs, provides opportunity for interactive application and feedback, and allows learners to engage with material over multiple timepoints to facilitate consolidation. It also addresses clinicians' desire for access to training materials for independent review and hands-on practice with the system (Supplementary Table 1). Training resources were iteratively developed by author JS in consultation with the knowledge translation lead (CP) and feedback from other team members. We piloted the training program with four individuals who had no prior *R2Play* experience to identify gaps from the perspective of novice learners. Materials were then revised to improve clarity and finalized.

**Figure 3:**
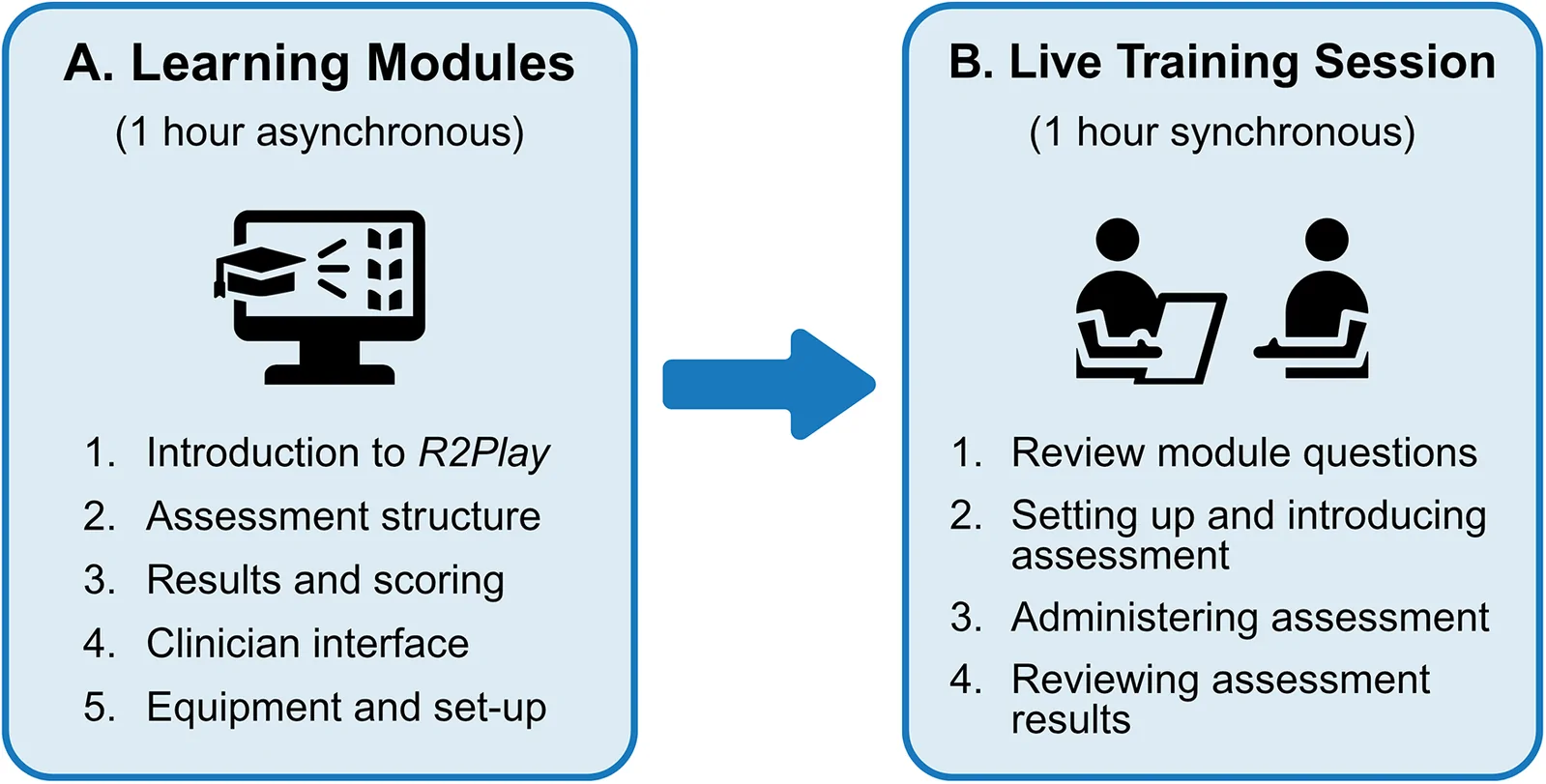
Overview of *R2Play* training program. Participants first complete learning modules **(A)**, accessed from a training hub webpage. Each module includes learning objectives, a didactic video, knowledge check questions, downloadable handouts, and key take-away messages. During the live training session **(B)**, participants apply knowledge gained through the modules by working directly with the *R2Play* system through a series of key tasks to practice setting up, administering, and reviewing results from an *R2Play* assessment.

Figure 4 provides an overview of resulting *R2Play* learning modules, which each include: (1) learning objectives; (2) didactic video; (3) knowledge check questions; (4) companion resources; and (5) key take-away messages. During live training, learners apply knowledge gained through the modules to practice *R2Play* administration skills, including system set-up, facilitating levels, and reviewing results. The live training task list is shown below in Figure 5.

**Figure 4:**
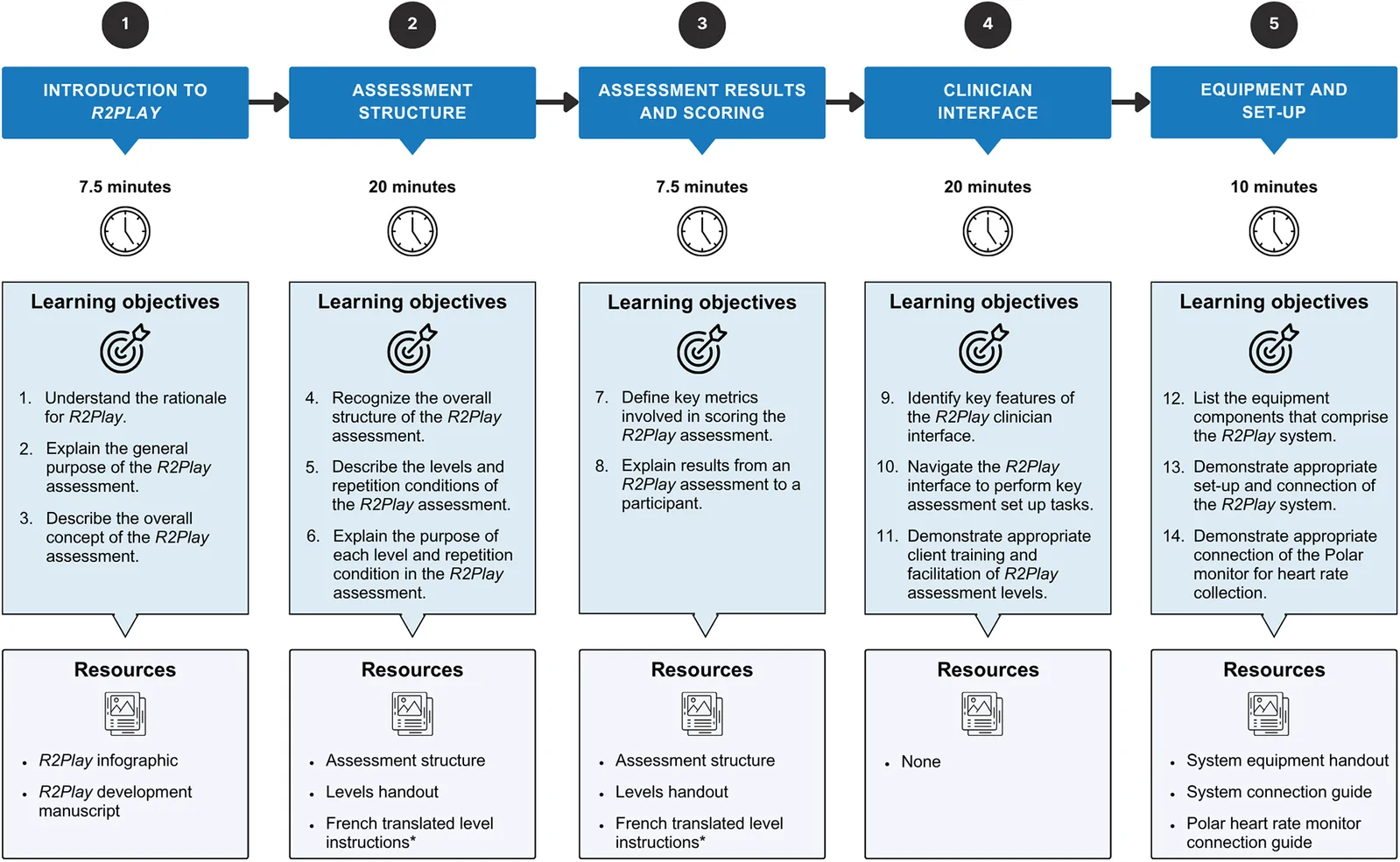
Overview of asynchronous *R2Play* learning modules (∼1 h total). *This handout was added partway through the study upon request from bilingual site participants.

**Figure 5:**
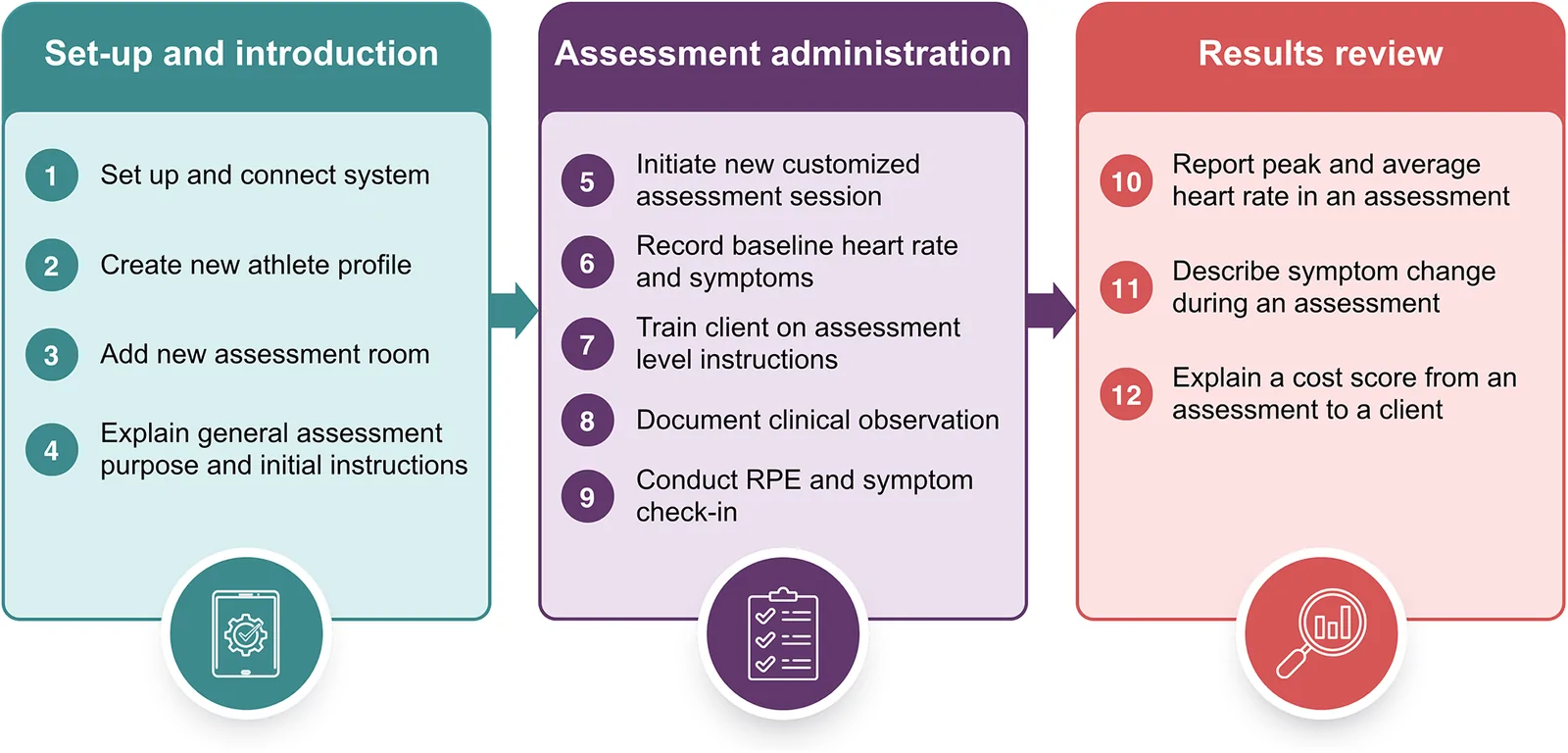
Key task list for live training simulation activity.

### Objective 2: training delivery

#### Step 5: training implementation

##### Participants

Clinician participants were recruited from staff at each site and the local community (42). Inclusion criteria: (1) registered or certified healthcare professional or learner, (2) experience working with youth or young adults, (3) experience working with concussion or other acquired brain injury population, and (4) fluent in English (or French at bilingual sites). Clinicians were excluded if they had previous experience administering *R2Play*.

##### Protocol

Upon enrolment, participants received an email providing training instructions and access to the learning modules webpage, which they were asked to complete before their live training session. During live training, participants were present in-person at their site working directly with *R2Play*. Training was facilitated by authors JS and AL in-person at the lead site and virtually for other sites, supported by local staff present in-person. Sessions began with an opportunity for questions about module information, followed by the practice activity to review key *R2Play* administration tasks. Standardized scripts were used to guide participants through tasks (Figure 5). Participants were encouraged to bring any notes from reviewing the modules and were given a copy of each written resource that they could reference and annotate. Researchers intervened as little as possible, allowing participants to attempt each task, assisting when needed, and then confirming task completion and providing feedback. All training activities were conducted in English.

### Objective 3: training evaluation

#### Step 6: training evaluation

A preliminary evaluation of the training program was conducted based on the Kirkpatrick Model for Training Evaluation (44), which includes four levels: (1) *Reaction*—learners' perceptions of training; (2) *Learning*—acquisition of intended knowledge, skills, confidence, attitudes, and commitments; (3) *Behavior*—application of learning in practice post-training; and (4) *Results*—long-term impact and outcome achievement. In the context of the broader pilot study, we conceptualized Level 3 (Behavior) as whether participants successfully applied their learning to administer *R2Play* independently during their two assessment sessions, which will be explored in a follow-up study. Level 4 (Results) considers organizational impacts and is therefore beyond scope of current work. Thus, initial evaluation activities in this study focused on Levels 1 and 2. Table 2 outlines the evaluation questions and measures, which are detailed below. A multi-method approach was used whereby both quantitative and qualitative data were collected, analyzed separately, and reported in parallel without explicit integration.

**Table 2:** R2Play training evaluation according to the kirkpatrick model for training evaluation (44).

Level	Components	Guiding questions	Evaluation
1. Reaction	Satisfaction	How favorable do learners find training? How satisfied are learners with the content and format of training?	Post-training survey Follow-up interview
	Relevance	How relevant is training content to learners' clinical experience? How well does the training format meet the needs of learners?	Post-training survey Follow-up interview
	Engagement	To what extent are learners actively involved in the training experience? Which elements helped engage learners? How interesting, compelling, or captivating do learners find training?	Post-training survey Follow-up interview Module knowledge check questions
2. Learning	Knowledge	To what extent do learners obtain the knowledge needed to deliver *R2Play*? How well does training convey the information needed to deliver *R2Play*? Which aspects of training helped learners develop knowledge of *R2Play*?	Post-training survey Follow-up interview
	Skills	To what extent do learners obtain the skills needed to deliver *R2Play*? How well does training teach the key tasks that comprise delivery of *R2Play*? Which aspects of training helped learners develop these skills?	Post-training survey Follow-up interview Key task practice activity
	Confidence	How confident are learners during their *R2Play* assessment sessions? To what extent do learners think they can successfully deliver *R2Play*? What contributed to learners' sense of confidence?	Post-training survey Follow-up interview

##### Evaluation measures

###### Participant characteristics

Participants completed a demographics form to capture relevant characteristics including age, gender, ethnicity, discipline, years of experience, practice setting, and general experience and comfort integrating technology within their practice.

###### Knowledge check questions

Each learning module included 4–6 knowledge check questions (true/false, multiple choice, or checkbox) as a formative assessment of learning engagement (Supplementary Table 5). Questions used a convergent style (single correct answer) scored as correct (1) or incorrect (0) (50). After submitting responses, participants received feedback on their score and correct answers. This allowed learners to reflect on information presented and confirm their understanding. Since participants had to actively pay attention and intentionally process module content to answer correctly, these questions provide evidence of engagement in learning, while also supporting participants' immediate comprehension of training material.

###### Post-training feedback survey

Participants completed a feedback survey after training that included specific questions for each component of the Kirkpatrick model. The survey captured participants' reactions to training in terms of satisfaction, relevance, and engagement (Kirkpatrick Level 1), as well as subjective perceptions of learning (knowledge, skills, and confidence) and self-reported perceived achievement of each learning objective (Kirkpatrick Level 2).

###### Live training key task checklist

To measure learning objectively (Kirkpatrick Level 2), participants' knowledge and skills in delivering *R2Play* were evaluated based on completion of key tasks during live training. Tasks (Figure 5) included original assessment administration activities defined in earlier study phases (39, 41) and newly added competencies (e.g., system set-up). Per previous work (39, 41), performance was scored for each task as:

2. Completed fully independently: Participant successfully completes the task in its entirety independent of any guidance from the research team.
1. Completed partially or with help: Participant partially completes the task (i.e., misses some details) or requires guidance from the research team.
0. Did not complete: Participant does not complete the task at all.

Notes were taken to capture any errors or assistance required. Both training facilitators (JS and AL) and local staff each rated task completion independently using standardized criteria (Supplementary Table 2) and compared scores. Discrepancies were rare and resolved through discussion to establish consensus on final scores. Total score was calculated as the sum of individual task scores, with a maximum score of 24.

###### Follow-up interviews

Semi-structured interviews were conducted virtually with participants within approximately one week after their last assessment session to obtain feedback on their experience with *R2Play* (42). Questions about training (Supplementary Table 3) were intended to elicit detailed perspectives by reflecting on their experience learning and delivering *R2Play* across multiple timepoints, including suggested improvements. Interviews were audio recorded, transcribed, and checked for accuracy.

##### Analysis

Knowledge check question accuracy was ascertained from the percentage of participants who answered correctly, and module averages were calculated. Survey responses and live training task completion scores were summarized descriptively using frequencies. Reasons for point deductions from task scoring were also grouped to identify common errors and areas requiring assistance.

Interviews were analyzed qualitatively using a hybrid content analysis (51) approach. Two authors (JS and BI) collaboratively coded a subset of transcripts to develop a preliminary codebook, which was then transferred to NVivo qualitative analysis software (Lumivero) and applied to remaining transcripts by the lead author (JS), while allowing flexibility for the addition of new codes to capture unique ideas. All codes were initially derived inductively to capture participants' perspectives in their own terms without being constrained by predefined theoretical constructs. Codes were then deductively organized around the Kirkpatrick Model constructs in Table 2 to facilitate meaningful interpretation of the data in relation to the study objectives and evaluation framework. This hybrid approach enabled us to integrate emergent insights that may have been missed through a purely deductive analysis and organize findings in a way that maintained alignment between research questions, theoretical concepts, and data sources.

## Results

### Overview

Across sites, 18 participants were trained over a 6-month period; 2 withdrew after training due to availability conflicts and did not complete *R2Play* assessment sessions or follow-up interviews. The sample primarily consisted of physiotherapists (61.1%) and athletic therapists (16.7%) who were white (77.8%) and identified as women (83.3%). There was diversity in participant ages, years of clinical experience, and history of technology use in practice (Table 3). Sites were anonymized throughout the results to protect participant privacy. Quantitative and key qualitative evaluation results are presented below according to the Kirkpatrick levels and guiding questions outlined in Table 2. A sample of qualitative analysis codes and quotes can be found in Supplementary Table 4.

**Table 3:** Participant characteristics by research site and total sample.

Characteristic, *n* (%)	Site 1 (*n* = 5)	Site 2 (*n* = 5)	Site 3 (*n* = 3)	Site 4 (*n* = 5)	Total (*n* = 18)
Practicing profession					
Physiotherapist (PT)	2 (40%)	3 (60%)	1 (33.3%)	5 (100%)	11 (61.1%)
Athletic therapist (AT)	3 (60%)	0	0	0	3 (16.7%)
Occupational therapist (OT)	0	1 (20%)	0	0	1 (5.6%)
Registered nurse (RN)	0	1 (20%)	0	0	1 (5.6%)
Medical doctor (MD)	0	0	1 (33.3%)	0	1 (5.6%)
Physiotherapy assistant (PTA)	0	0	1 (33.3%)	0	1 (5.6%)
Age range (years)					
20–30	2 (40%)	2 (40%)	0	2 (40%)	6 (33.3%)
30–40	2 (40%)	2 (40%)	2 (66.7%)	0	6 (33.3%)
40–50	1 (20%)	1 (20%)	1 (33.3%)	1 (20%)	4 (22.2%)
50+	0	0	0	2 (40%)	2 (11.1%)
Gender					
Man	2 (40%)	1 (20%)	0	0	3 (16.7%)
Woman	3 (60%)	4 (80%)	3 (100%)	5 (100%)	15 (83.3%)
Ethnicity					
White	4 (80%)	3 (60%)	2 (66.7%)	5 (100%)	14 (77.8%)
Filipino	1 (20%)	0	0	0	1 (5.6%)
Arab	0	1 (20%)	0	0	1 (5.6%)
Chinese	0	1 (20%)	0	0	1 (5.6%)
West Asian	0	0	1 (33.3%)	0	1 (5.6%)
Years of experience					
0–3	1 (20%)	1 (20%)	0	2 (40%)	4 (22.2%)
3–5	1 (20%)	2 (40%)	0	0	3 (16.7%)
5–10	1 (20%)	1 (20%)	1 (33.3%)	0	3 (16.7%)
10+	2 (40%)	1 (20%)	2 (66.7%)	3 (60%)	8 (44.4%)
Practice setting					
Public	1 (20%)	4 (80%)	1 (33.3%)	3 (60%)	9 (50.0%)
Private	3 (60%)	1 (20%)	2 (66.7%)	0	6 (33.3%)
Mixed	1 (20%)	0	0	2 (40%)	3 (16.7%)
Frequency of technology use in practice*					
Never	1 (20%)	0	0	1 (20%)	2 (11.1%)
Rarely	2 (40%)	3 (60%)	1 (33.3%)	3 (60%)	9 (50.0%)
Somewhat often	1 (20%)	1 (20%)	0	1 (20%)	3 (16.7%)
Very often	1 (20%)	0	2 (66.7%)	0	3 (16.7%)
All the time	0	1 (20%)	0	0	1 (5.6%)
Comfort using technology in practice					
Not at all comfortable	0	0	0	0	0
Slightly comfortable	1 (20%)	0	0	0	1 (5.6%)
Somewhat comfortable	3 (60%)	3 (60%)	2 (66.7%)	4 (80%)	12 (66.7%)
Very comfortable	1 (20%)	1 (20%)	1 (33.3%)	1 (20%)	4 (22.2%)
Extremely comfortable	0	1 (20%)	0	0	1 (5.6%)
Days from live training to first *R2Play* assessment session**					
0–15	2 (40%)	1 (20%)	0	0	3 (18.8%)
15–30	1 (20%)	1 (20%)	0	0	2 (12.5%)
30+	2 (40%)	3 (60%)	1 (33.3%)	5 (100%)	11 (68.8%)
Days between *R2Play* assessment sessions**					
0–15	5 (100%)	2 (40%)	1 (33.3%)	5 (100%)	13 (81.3%)
15–30	0	1 (20%)	0	0	1 (6.25%)
30+	0	2 (40%)	0	0	2 (12.5%)

* Participants were asked the following question: “How often do you use technology in your clinical practice for therapeutic purposes?” This includes but is not limited to mobile applications (e.g., Neurovine, Switched on Training), perceptual-cognitive training systems (e.g., NeuroTracker), computerized cognitive assessment and training platforms (e.g., ImPACT), smart watches (e.g., Fitbit), wearable inertial sensors for balance and gait assessment (e.g., AX6), and exercise gaming systems (e.g., X-Box Kinect, Nintendo Wii).

** Two participants from site 3 withdrew before assessment sessions (*n* = 16).

### Kirkpatrick level 1: reaction

#### Satisfaction

Ninety-four percent of participants rated “agree” or “strongly agree” to all three satisfaction questions in the feedback survey (Table 4), indicating a high level of satisfaction with the training program, and that the training content and format were appropriate.

**Table 4:** Post-training feedback survey results.

Question	Responses, *n* (%)				
	Strongly disagree	Disagree	Neither agree nor disagree	Agree	Strongly agree
*Satisfaction*					
I was satisfied with the training program overall.	0	0	1 (5.6%)	11 (61.1%)	6 (33.3%)
The content of the training program was appropriate to teach me the *R2Play* assessment.	0	0	1 (5.6%)	8 (44.4%)	9 (50.0%)
The format of the training program was appropriate to teach me the *R2Play* assessment.	0	0	1 (5.6%)	10 (55.6%)	7 (38.9%)
*Relevance*					
The information provided in the training program was appropriate for my level of clinical experience.	0	0	2 (11.1%)	7 (38.9%)	9 (50.0%)
The information provided in the training program was relevant to my clinical practice.	0	1 (5.6%)	3 (16.7%)	6 (33.3%)	8 (44.4%)
*Engagement*					
The information presented in the training program was easy to follow.	0	0	3 (16.7%)	7 (38.9%)	8 (44.4%)
The training program retained my interest.	0	3 (16.7%)	2 (11.1%)	5 (27.8%)	8 (44.4%)
*Knowledge*					
The training program gave me the knowledge I need to deliver the *R2Play* assessment independently.	0	1 (5.6%)	3 (16.7%)	7 (38.9%)	7 (38.9%)
*Skills*					
The training program gave me the skills I need to deliver the *R2Play* assessment independently.	0	0	2 (11.1%)	9 (50.0%)	7 (38.9%)
*Confidence*					
I am confident that I can successfully deliver the *R2Play* assessment independently.	0	3 (16.7%)	3 (16.7%)	6 (33.3%)	6 (33.3%)

Favorability was also captured in the interviews, with training commonly described as “good” or “great” and “done well” or “worked well”. Participants found the online modules straightforward and appreciated the embedded learning objectives, takeaway messages, and review questions. The companion resources were specifically highlighted as helpful for following system set-up and assessment steps and taking notes for reminders. For example, one participant explained, “The handouts were very helpful… one of the most useful tools that we got because we can just carry those around until we learn it… like any other assessment we use in clinic, we carry little cheat sheets until we figure it out” (PT_4_, Site 4). Most participants felt they received enough training, saying it was “thorough”, “comprehensive”, “sufficient”, and “adequate”. Nevertheless, recommendations for additional content included familiarization with the software interface, addressing unexpected situations, troubleshooting technical issues, and interpreting results.

#### Relevance

While most participants (88.9%) agreed or strongly agreed that the information provided in the training program was *appropriate* for their level of clinical experience, some responded neutrally (16.7%) or disagreed (5.6%) that the information was *relevant* to their clinical practice (Table 4).

Interviews provided limited insight into the relevance of training *content* to clinical practice, with two participants expressing a need for more contextual background on current best practices in concussion management and return-to-play assessment for clinicians with limited experience. Conversely, many participants spoke about the appropriateness of the training *format* for their learning needs as clinicians. Specifically, they appreciated the multi-pronged training approach (videos, written resources, hands-on practice) that satisfied diverse learning styles, as well as the self-paced and segmented nature of learning modules, making them easier to complete within busy clinical schedules. For example, one participant commented on how combining didactic and applied learning aligns with clinical education practices:

“In terms of medical or healthcare education overall, this is how we do it… we go through an online lecture portion, understand the basics and the foundation of what”s going on, and then we actually practice them one time in person, like a lab portion..So having a system do that I think is very familiar to me.”—*PT_3_, Site 2.*

The training program was generally viewed as time-efficient, though some participants felt that modules should be shortened to retain learners' attention and enable more hands-on practice.

#### Engagement

Responses to knowledge check questions were generally accurate with at least two-thirds (66.7%) of participants answering correctly on all but one question (Figure 6), indicating good engagement with learning module content. Accuracy was lowest for *Module 3: Assessment results and scoring* (mean = 80.6% ± 16.0%) compared to other modules (mean 86%–90%).

**Figure 6:**
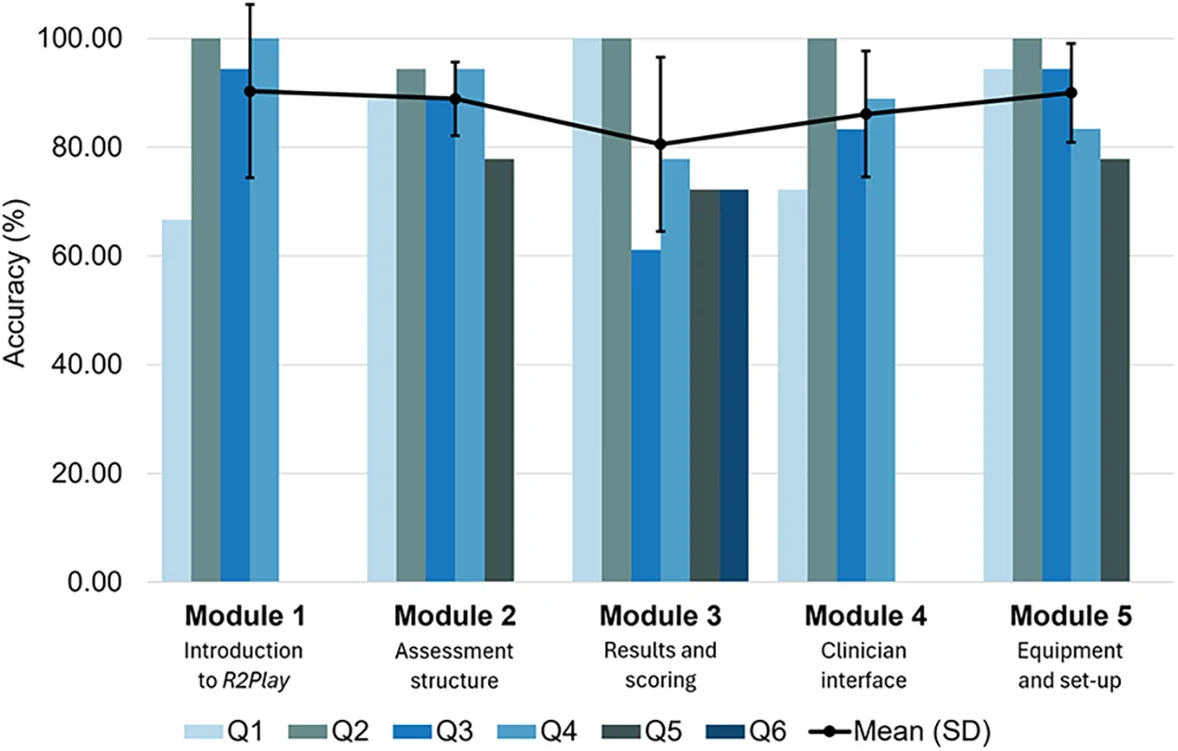
Knowledge check question accuracy by module. Accuracy refers to the percentage of participants who answered the question correctly. Question content can be found in Supplementary Table 5.

In the post-training survey, most participants (83.3%) agreed that the information presented was easy to follow, however, almost one third (27.8%) responded neutrally or disagreed that the training program retained their interest (Table 4). Interviews reinforced that modules were easy to follow and revealed various ways that participants engaged with training materials to become active learners. For example, one participant mentioned taking notes while watching modules:

“I would write some notes here and there, as you were talking… you have your video, you stop it, you look at your handout, you're able to write things… So, I was able to follow in the video but also stop it to take a few notes to make sure I understood.”—*PT_2_, Site 4*.

Several participants reported that they re-reviewed training modules or handouts *before* assessment sessions to refresh their memory or address uncertainties and appreciated having retained access to materials. Some also mentioned referencing handouts *during* sessions to help follow assessment procedures. Lastly, Site 4 participants commented on how collegial discussion within their team supported learning: “As a group, we did exchange on our specific experiences, which did help. If you were the only person in [a] clinic to run this and you had no one to troubleshoot with, [it] could be a little bit challenging” (*PT_1_, Site 4*).

### Kirkpatrick level 2: learning

#### Knowledge and skills

Participants performed well in the training task activity (Table 5), with a median total score of 19/24 (79.2%) and range of 16–21 (66.7%–87.5%). Participants required most assistance with setting up the system (77.8%), explaining the purpose and instructions (100%), training clients on a level (94.4%), and explaining cost score results (72.2%). In the survey, most participants agreed that they gained the knowledge (77.8%) and skills (88.9%) to deliver *R2Play*.

**Table 5:** Live training key task activity scoring results.

Task	Scores, *n* (%)			Top reasons for point deductions*
	Completed fully independently (2)	Completed partially or with help (1)	Did not complete (0)	
1. Set up and connect the *R2Play* system	4 (22.2%)	14 (77.8%)	0	Order of set-up steps (*n* = 8) Equipment placement or configuration (*n* = 8) System connection (*n* = 5)
2. Create athlete profile	18 (100%)	0	0	Difficulty navigating clinician interface (*n* = 1)
3. Add new assessment room	17 (94.4%)	1 (5.6%)	0	N/A
4. Explain general purpose and instructions for *R2Play*	0	17 (94.4%)	1 (5.6%)	Assessment concept or instructions (*n* = 15) Assessment purpose (*n* = 5)
5. Begin new assessment session with specified customization	18 (100%)	0	0	N/A
6. Administer baseline assessment of heart rate and symptoms	12 (66.7%)	6 (33.3%)	0	Premature termination of HR connection (*n* = 4) Failure to complete HR connection (*n* = 2)
7. Train new client on a given level of the assessment	1 (5.6%)	17 (94.4%)	0	Missed instruction details (*n* = 9) Failure to introduce assessment sounds (*n* = 8) Did not use video demonstration or tracing practice function (*n* = 5)
8. Document clinical observation during an assessment	18 (100%)	0	0	N/A
9. Conduct check-in assessment of RPE and symptoms	15 (83.3%)	3 (16.7%)	0	Did not complete symptom check in (*n* = 2) Assistance from local site research staff (*n* = 1)
10. Report peak and average heart rate results	17 (94.4%)	1 (5.6%)	0	Locating value within interface (*n* = 1)
11. Describe change in client's symptoms during an assessment	10 (55.6%)	8 (44.4%)	0	Locating value within interface (*n* = 8)
12. Explain cost score from an assessment to a new client	5 (27.8%)	12 (66.7%)	1 (5.6%)	Level/condition comparison (*n* = 5) Direction of cost score comparison (*n* = 3) Explaining concept of cost score (*n* = 3) Locating correct value within interface (*n* = 2)

* Counts for point-deduction reasons may exceed task scoring totals if multiple reasons were coded per participant.

Interview data suggested that learning modules were a useful introduction to important foundational information before attempting to use *R2Play*. For example, one participant said, “The modules first I think was a good intro… it was a great way to just learn about the concepts and learn about what is *R2Play* and what it does” (RN_1_, Site 2). Five participants highlighted the knowledge check questions as helpful for detecting knowledge gaps and reinforcing learning. Some participants reported trouble understanding *R2Play* instructions from the modules or found it difficult to follow discrete videos presenting each assessment component separately without showing the full picture; An end-to-end demonstration of the entire assessment workflow was recommended to help consolidate information, especially for visual learners.

“Learning by doing” through hands-on practice was generally regarded as most critical to fully understand how *R2Play* works and become familiar with the system before using it with clients. Many participants described how seeing the system in action and practicing key tasks helped “put it all together” by solidifying module information, clarifying uncertainties, and learning administration processes (e.g., set-up, level instructions). With feedback from the trainers, either virtually or in-person, participants learned from mistakes and improved their delivery: “It was nice to have the one-on-one and you guys being there, kind of evaluating me and giving your feedback on what I missed and how maybe I could change my explanation” (PT_3_, Site 4). To further enhance learning, several participants expressed a preference to complete a full mock run-through of the entire assessment, rather than only practicing key tasks individually. Watching experienced users deliver *R2Play*, through shadowing opportunities or recorded videos, was also recommended to help learners understand proper administration techniques.

#### Confidence

In the post-training survey, two thirds (66.7%) of participants agreed or strongly agreed that they were confident they could successfully deliver *R2Play* independently. Participants largely agreed that they achieved learning objectives (Table 6), with the lowest agreement for explaining assessment results to a participant (72.2%).

**Table 6:** Self-reported perceived achievement of learning objectives. .

Learning objectives	Responses, *n* (%)				
	Strongly disagree	Disagree	Neither agree nor disagree	Agree	Strongly agree
1. I understand the rationale for *R2Play*.	0	0	0	6 (33.3%)	12 (66.7%)
2. I can explain the general purpose of the *R2Play* assessment.	0	0	0	6 (33.3%)	12 (66.7%)
3. I can describe the overall concept of the *R2Play* assessment.	0	0	0	5 (27.8%)	13 (72.2%)
4. I can recognize the overall structure of the *R2Play* assessment.	0	1 (5.6%)	0	6 (33.3%)	11 (61.1%)
5. I can describe the levels and repetition conditions of the *R2Play* assessment	0	1 (5.6%)	0	10 (55.6%)	7 (38.9%)
6. I understand the purpose of each level and repetition condition in the *R2Play* assessment.	0	0	0	10 (55.6%)	8 (44.4%)
7. I can define key metrics involved in scoring the *R2Play* assessment.	0	0	2 (11.1%)	11 (61.1%)	5 (27.8%)
8. I can explain results from an *R2Play* assessment to a participant.	0	0	5 (27.8%)	9 (50.0%)	4 (22.2%)
9. I can identify key features of the *R2Play* clinician interface.	0	0	2 (11.1%)	9 (50.0%)	7 (38.9%)
10. I can navigate the *R2Play* interface and perform key assessment set-up tasks.	0	0	2 (11.1%)	8 (44.4%)	8 (44.4%)
11. I can demonstrate appropriate client training and facilitation of *R2Play* assessment levels.	0	0	1 (5.6%)	12 (67.7%)	5 (27.8%)
12. I can list the equipment components that comprise the *R2Play* system.	0	0	0	8 (44.4%)	10 (55.6%)
13. I can demonstrate appropriate set-up and connection of the *R2Play* system equipment.	0	0	0	10 (55.6%)	8 (44.4%)

When interviewed, participants generally reported feeling well-prepared and confident delivering *R2Play* after training, while recognizing that adapting to new technology takes time. Indeed, five participants explicitly mentioned having more confidence in their second *R2Play* session. For some, lengthy delays (>30 days) between training and subsequent study sessions were thought to have reduced information retention and lowered confidence. Consistent with survey data, results interpretation was described as the topic of least comfort that would require more experience. System set-up and connection were also initially stressful for some participants, but the process got easier with practice using the set-up guides, and participants realized it was less complicated than they first thought. Across the sample, nearly all participants emphasized that support from an experienced user (i.e., research staff) made them feel more comfortable when initially using *R2Play* and could be an important facilitator of clinical implementation. For example, one said:

“Talking through things with [research staff], being able to ask questions was helpful… I could see that being done in a clinic where someone the first time they're doing it has a clinician [who] has run it before, just to ask questions and show them.”—OT_1_, Site 2

## Discussion

This study developed, delivered, and evaluated a clinician training program for *R2Play*, a novel technology-based concussion assessment tool*.* The training program, which involved learning modules and a live practice session, was delivered to 18 clinicians across four sites, with feedback generally indicating positive reactions and learning perceptions. Preliminary evaluation results showed that participants objectively and subjectively experienced most difficulty learning system set-up, delivering precise level instructions, and explaining scoring metrics. Interviews highlighted topics for additional content (e.g., navigating software interface, interpreting results, managing technical issues) and suggested improvements, including end-to-end demonstrations, mock run-through of the full workflow, and opportunities to watch experienced users deliver the assessment. This work shows how early focus on user training can help inform the design and implementation of DHTs.

### Implications for *R2Play* and the concussion field

Evidence across evaluation measures highlights challenges participants faced in learning *R2Play* scoring. This is consistent with our proof-of-concept and feasibility studies (40, 41) that highlighted clinicians' need for support to apply cost score metrics. Apprehension toward multidomain cost scoring is unsurprising, as it is a significant departure from current concussion assessment practices that predominantly focus on symptom reports and single-domain measures (e.g., exertion testing, balance, cognition) (52). Moreover, the psychometrics and significance of *R2Play* scores remain unknown at this stage, which likely adds to discomfort. Nevertheless, participants struggled with conceptually *explaining* cost scores, which intentionally avoided *interpretation* or *application* that would warrant validation data. Reasons for difficulty included confusion about level comparisons, directionality, and navigating the *R2Play* interface (Table 5), suggesting room for improvement with more experience and/or improved user interfaces. Future work evaluating participants' performance during subsequent assessment sessions (Kirkpatrick level 3) would help understand the learnability of cost scoring. As complex concussion assessments like *R2Play* mature with more evidence, other learning methods (e.g., case-based) (53) may be needed to train clinical reasoning skills for interpretation and application in practice. These findings underscore the importance of accessible scoring for these novel assessments (38) and provide useful learnings for training initiatives to build clinicians' capacity for new concussion assessments methods.

Despite favorable ratings for information clarity and appropriateness for participants' experience, lower scores were observed for retention of interest and relevance to practice. This could reflect the study context wherein participants were not expected to change practices, a typical goal of training (44). Our sample also includes clinicians from diverse disciplines, settings, and roles who may not be positioned to use *R2Play* or influence return-to-play decisions. Knowles' model of adult learning (Andragogy) offers useful strategies to enhance interest and relevance (54). According to Andragogy, adults are motivated to learn when they understand the *need* for learning and its relevance to solving real-life problems (54). While our curriculum focused on knowledge and skills to *administer R2Play*, more background on its clinical purpose and scientific rationale could make training more relevant for clinicians. Indeed, two participants suggested more context on current practices. Andragogy also advocates self-direction and problem-based methods whereby adults are responsible for their own learning and integrating new information with previous experience for real-world application (54). These principles may be addressed through active learning activities (e.g., simulation, discussion, case study) and self-selected components, including those suggested by participants (e.g., “free-play” interface, video demonstrations, shadowing).

### Implications for DHT training and implementation

Despite its importance for implementation (10, 15), little is known about *how* to train clinicians on DHTs (55). While implementation requires a context-tailored approach, this study adds to limited empirical literature on considerations for DHT training content and format in rehabilitation settings. In a qualitative study of clinician experiences integrating technologies in rehabilitation practice, Pearce et al. found practical hands-on training most useful, including guidance for device set-up and supervised practice (56). This aligns with our work wherein participants emphasized “learning by doing” and receiving feedback as critical for developing competence in *R2Play*. Our findings are also consistent with Pearce et al. on the need for training to address skills beyond technical proficiency (e.g., communication) (56), as our participants had difficulty articulating the purpose of *R2Play* and delivering its instructions. While more robust evidence will help facilitate these conversations, additional strategies may be needed to prepare clinicians for communicating about DHTs. This could include scripts, modeling, or roleplay. Based on our experience, Table 7 provides evidence-based practical recommendations for training clinicians on DHTs in rehabilitation, which are also presented as an infographic in Supplementary Image 1. Future research is needed to explore the efficacy and efficiency of these training strategies (55).

**Table 7:** Recommendations for training clinicians on DHTs in rehabilitation.

Recommendation	Description
1. Include background context.	Introduce the tool by describing its purpose and rationale within the context of current practices.
2. Train competencies in technical, clinical, and communication skills (*as applicable*).	Depending on the nature and purpose of the DHT, consider designing training components to address skills related to: Technical proficiency: Equipment setup, system connection, navigating software interface, managing technical issues. Clinical reasoning: Understanding outputs and metrics, interpreting data, applying to clinical decision-making. Communication: Introducing the tool to patients/families, delivering standardized instructions, and explaining results.
3. Use a blended, multi-modal training format.	Use a combination of asynchronous learning (e.g., online modules, videos, manuals) and hands-on practice activities. Deliver material through multiple modes to accommodate diverse learning styles, including written information (e.g., manuals, handouts, etc.), visual elements (e.g., diagrams, videos), auditory explanations and experiential activities.
4. Prioritize hands-on practice.	Ensure direct interaction with the technology during training. Consider structured task-based simulations. Provide real-time feedback on learners' performance.
5. Consider the full clinical workflow.	Ensure that training covers the entire clinical workflow (e.g., setting up equipment, connecting system, configuring settings) Provide an end-to-end demonstration of the full workflow. Allow learners to complete a practice run of the full workflow.
6. Provide structured scripts and guides.	Provide step-by-step set-up guides, administration scripts, and troubleshooting resources for quick reference. Encourage clinicians to use these aids during training practice sessions and early implementation.
7. Facilitate ongoing review and practice.	Enable ongoing access to training materials and encourage learners to re-review them as needed. Provide access to practice environments (e.g., sandbox or “free play” version) and time for self-directed exploration. Align the timing of training as close to clinical use as possible to limit knowledge decay and offer booster sessions when possible
8. Build capacity for troubleshooting.	Include training on how to address common technical errors. Provide resources for managing unexpected scenarios.
9. Incorporate shadowing and mentorship.	Provide opportunities to observe experienced users model administration (either in person or through recorded videos). Incorporate mentorship and feedback from experienced users.
10. Promote collaborative learning activities.	Provide opportunities for peer-to-peer learning and interaction. Encourage group reflection, discussion, and debriefing.

Beyond initial training, supportive organizational initiatives can help inspire sustained clinician confidence in DHTs (9, 10). According to Pearce et al., clinicians develop confidence in rehabilitation technologies through two stages, first learning basic set-up and device operation, then advanced clinical reasoning (56). Confidence was generally high in our sample but only reflects the first stage of learning *R2Play* delivery since clinical application was beyond scope. Peer leadership and collaboration are partly responsible for building this confidence. Local staff played an important role as “champions” who leveraged their own *R2Play* experiences and contextual knowledge to mentor participants, support them through learning discomfort, and help them navigate emergent issues (e.g., technical issues) (49). Indeed, champions are crucial for DHT implementation by persuading peers, facilitating clinical integration, and troubleshooting technical issues (9, 56). In addition, feedback from Site 4 participants highlighted the benefits of collegial discussion and collaborative team learning, while participants at other sites were unknown to each other and had no opportunity for interaction. As clinicians value peer learning and support (56), interaction should be facilitated during training (e.g., case studies, group discussion, simulations) and beyond using methods such as clinical rounds, learning collaboratives (57) and communities of practice (58).

### Benefits of early multicenter training evaluation

Developing and evaluating training materials with clinician end-users through early cross-site feasibility testing had several benefits for *R2Play* with respect to issues listed in Table 1. First, uncovering critical user issues (delivering instructions, explaining results) led to substantial re-designs and addition of new support features (scripts, embedded explanations, visualizations). Second, formal training created a realistic study experience for participants, producing practice-oriented feedback (e.g., “*I could see that being done in a clinic*…”) that helps address broader feasibility aims (42) and informs future planning for implementation strategies beyond training (clinical champions, learning collaboratives) (49). Finally, standardized training and criteria for *R2Play* competency and assessment quality will enable further clinician involvement in future work and support measurement fidelity to ensure the integrity of validation study findings (33, 42).

### Limitations and future directions

Although inclusion of multiple sites and evaluation methods are strengths of this study, there are limitations to our sample and data. All sites were academic-affiliated, multidisciplinary research clinics, and there was limited diversity in clinician disciplines, genders, and ethnicities, making the generalizability of findings to other groups and settings unclear. We did not collect information on participants' previous concussion education, which could affect training needs. Although response rates were excellent, evaluative measures were administered by the training facilitator and *R2Play* design team, which risks response favorability biases in survey and interview data. Additionally, surveys and interviews were not fully aligned in timing and content due to broader study constraints; surveys were completed immediately post-training, while interviews occurred after participants' last *R2Play* session (many days later) and included only brief questions on training that differed from the survey. This may have caused recall bias in interview data and limited deeper mixed methods integration (59). Some participants reported feeling less competent and/or confident due to delays between training and assessment sessions, however temporal effects were not directly evaluated and should be explored further. We were also unable to determine whether language of instruction influenced training outcomes for bilingual participants.

This study took place within a research context, without opportunity for practice changes, and findings may not fully translate to real-life clinical settings. Initial evaluation data only addressed levels 1 (reaction) and 2 (learning) of the Kirkpatrick model; future work on level 3 (behavior) should explore learning application by assessing the quality of *R2Play* assessments administered post-training (44). Once *R2Play* matures, longer-term directions could include (1) planning implementation strategies beyond training; (2) evaluating the impact of training and other implementation strategies on *R2Play* adoption; and (3) determining the impact of *R2Play* training and implementation on clinical and organizational outcomes (Kirkpatrick level 4).

## Conclusions

This initial evaluation found positive reactions and learning perceptions among clinicians who were trained on a technology-based concussion assessment tool through online modules and a synchronous practice session. Participants had trouble learning system set-up, delivering precise instructions, and explaining scoring metrics, which suggests that DHT training should address technical, clinical, and communication skills. Findings demonstrate the value of early multicenter studies involving end-user training to enhance the usability of DHTs and identify other potential implementation strategies (e.g., clinical champions, learning collaboratives) that could help maximize the uptake and impact of DHTs.

## Data Availability

The original contributions presented in the study are included in the article/Supplementary Material, further inquiries can be directed to the corresponding author.
