# Supplementary material for: Designing for implementation: development, delivery, and multi-method evaluation of a clinician training program for a novel technology-based concussion assessment tool
Source: Front Health Serv. 2026 Oct 1;6:1937910. doi: 10.3389/frhs.2026.1937910 (PMC13669162; doi:10.3389/frhs.2026.1937910)
Supplement: Supplementary file 1 [file Image1.pdf]

# 10 Tips for training clinicians on digital health tools

These recommendations are drawn from a research study that developed and evaluated a clinician training program for a novel technology-based concussion assessment tool called *R2Play*. They summarize key lessons for training clinicians on digital health technologies in rehabilitation.

## 1 Include background context

Introduce the tool by describing its purpose and rationale within the context of current practice

## 2 Target skills for practice readiness

Consider the need for training to address skills related to technical proficiency, clinical reasoning, and communication with care recipients

## 3 Use a blended learning format

Deliver material through multiple modes to satisfy diverse learning styles, including written text, visual depictions, and auditory explanations

## 4 Prioritize hands-on practice

Maintain direct interaction with the technology during training and give feedback on performance

## 5 Consider the full clinical workflow

Ensure that training activities cover the full clinical workflow from equipment set-up and configuration through analysis and client debriefing

# 10 Tips for training clinicians on digital health tools

## 6 Provide structured resources

Offer step-by-step guides and scripts for quick reference during training and early implementation

## 7 Facilitate ongoing review and practice

Enable ongoing access to the digital tool and training materials for self-directed learning, and offer booster sessions to minimize knowledge decay

## 8 Build capacity for troubleshooting

Provide guidance and support resources for how to address technical errors and unexpcted scenarios

## 9 Incorporate shadowing and mentorship

Create opportunities to observe experienced users and receive mentorship and feedback

## 10 Promote collaborative learning activities

Support growth through peer-to-peer and/or group reflection, discussion, and debriefing

**Reference**: Shore J, Provvidenza C, Lovell A, Christopoulos E, Hickling A, Hutchison M, Munce S, Schneider KJ, Zemek R, Gagnon I, Biddiss E, Scratch SE. *Designing for implementation: Development, delivery, and multi-method evaluation of a clinician training program for a novel technology-based concussion assessment tool*. Submitted to *Frontiers in Health Services* Jul 14, 2026.
