# Supplementary material for: Designing for implementation: development, delivery, and multi-method evaluation of a clinician training program for a novel technology-based concussion assessment tool
Source: Front Health Serv. 2026 Oct 1;6:1937910. doi: 10.3389/frhs.2026.1937910 (PMC13669162; doi:10.3389/frhs.2026.1937910)
Supplement: Supplementary file 2 [file Table1.docx]

**Supplementary Table 1.** Perceived training needs from feasibility study.

| **Category** | **Description** | **Sample quotes** |
| --- | --- | --- |
| Multimodal training approach | The multimodal nature of the training session, comprised of didactic information, video demonstrations, and practice activities, was found to be engaging and effective. | "I thought you did a good job of having multiple modes, so it was auditory, there was visual, there were videos, you could interact. So, I think that was fun."  “I liked kind of the balance of just kind of getting information told to me, but then also the visual interface and then me having to actually try it with you acting as kind of the participant was helpful because it forced me to actually physically navigate through the interface itself.” |
| Interactive practice activity | The interactive practice activity was particularly useful for clinicians’ learning since it helped familiarize them with navigating the *R2Play* software interface, which made them more comfortable administering the assessment. | “I found the helpful part of the training, I think… is when I took over your screen as well because then I was like, “Okay, I know you said this, but I don't know where to click.” So, I think that was helpful.”  “Taking over your screen was actually really helpful and like making somebody actually do it, because I think if I was just doing it today, I would have been like [noise of discomfort]. So, I actually think that was really helpful.”  “... then having to actually try it with you acting as kind of the participant was helpful because it forced me to actually physically navigate through the interface itself.” |
| Demonstrations | Watching an experienced user administer the *R2Play* assessment could support clinicians’ learning by demonstrating appropriate delivery of level instructions. | “I think I would have… I personally would have learned better by watching someone else do it.”  “But even in the training like- whether it's you cue the people to be like, “hey, this is where it says your instructions,” or you know, you like, read them an example of how you would explain it. Obviously doesn't need to be like standardized or anything right now, but that in the training might be helpful or even just like a little… like, ‘golden nuggets’ kind of thing.” |
| Shadowing and feedback | When clinicians first learn to use *R2Play,* an experienced colleague could shadow their assessment to address any challenges that arise and provide feedback on their delivery approach to identify areas for improvement. | “I think if you were actually doing [*R2Play*] with your colleagues and practicing on it, there would be some sort of like discussion about what went well, what was difficult, how to improve, things like that... I think that's just more of like doing good clinical care.”  “I guess the first couple times, having someone who is well-experienced in *R2Play* to be there just in case there's any troubleshooting that needs to be done, and they can also shadow them and see if there's any improvements that need to be done as well, to make it go more smoothly.” |
| Access to training materials | Having access to asynchronous training materials (e.g., summary handouts and videos) for review prior to administering the assessment with a participant could serve as a helpful reminder of key information. | “Those handouts that you had available might be helpful for the clinician to just have on-hand to be able to review in advance.”  “Training-wise, I mean if there were like short little video snippets to remind me what to do, that would be something I would maybe like watch before I was to run through if I was doing it on my own.”  “I wonder if there would be some way to have like a… like I like the cheat sheet kind of component that's there, but that might be helpful even just to send through the clinician after, of “here's kind of the key points of what we discussed. If you want to review this before you come in” that could be helpful.” |
| Independent practice | In addition to formal training sessions, opportunities for independent practice with the *R2Play* system could help improve familiarity and comfort level delivering the assessment. | “I think it would be really cool to have like a practice interface that you can have access to at any time so.”  “If you were to take it from a research setting to a clinical setting… I mean you would get the tablet before you'd see the patients anyway, so you'd have a chance to interact with the interface a little bit more on your own, which I think would be a helpful part of training.”  “In terms of improving the training… it could even just- I guess in this stage it's hard to give somebody a way to practice almost on their own, or like, build in more time to just do whatever you want with the system could be helpful. Just so it's more like learning by doing it, because I know we only had like 10 minutes or so to do that.” |
| Participatory experience | Completing *R2Play* as a participant is a potentially valuable exercise for clinician learners as part of training to provide first-hand experience of the assessment to draw on when administering it. | “…also being the trainee to kind of just do it yourself… when I was training for certain things as a [kinesiologist] personally, the tests that we did, we also complete it ourselves just so we can understand how it feels, how tired you are, what could be challenging, so you can just have that as an experience.” |
| Troubleshooting common errors | Troubleshooting resources could help clinicians independently manage common errors that may arise during an *R2Play* assessment without external assistance. | “I think in terms of the software… if there is any sort of troubleshooting, how do you troubleshoot that? Because I didn't have to troubleshoot anything but like if I didn't have you guys there and something did go wrong, like I need some help just to troubleshoot, so if there's a manual, just like, “if this goes wrong you can do this. If this goes wrong, you can do this.” |
| Timing of training | The timing of training in relation to actual system use is an important factor for effective learning, as completing training close to the date of use would minimize the likelihood of forgetting key information. | "Just having it closer to like that start date, that's like the main thing just so it's not too rusty in my head."  “I don't know if it's maybe beneficial to, like, do the training right before you're about to do the session... I just think the time in between without putting it into practice and then the first time you're practicing is doing an assessment, was maybe a little bit harder.” |

*Note*. Quotes have been collated from unpublished data associated with the *R2Play* feasibility study under Holland Bloorview Kids Rehabilitation Hospital Research Ethics Board protocol #20‐099.
