# Supplementary material for: Designing for implementation: development, delivery, and multi-method evaluation of a clinician training program for a novel technology-based concussion assessment tool
Source: Front Health Serv. 2026 Oct 1;6:1937910. doi: 10.3389/frhs.2026.1937910 (PMC13669162; doi:10.3389/frhs.2026.1937910)
Supplement: Supplementary file 3 [file Table2.docx]

**Supplementary Table 2.** Evaluation criteria for live training key task completion.

| **Task** | **Evaluation criteria** |
| --- | --- |
| *Setting up and introducing assessment* | |
| 1. Set up and connect the *R2Play* system to prepare for an assessment session | - Lays out all equipment (router, tablets, speaker) in the appropriate positions as indicated in the *R2Play* system equipment handout - Successfully connects the system by following process outlined in the 7-step system set-up guide handout |
| 2. Create an athlete profile for a new client | Navigates to the client library and successfully creates a new athlete profile by entering the participant ID and age.  *Other sections of the athlete profile need not be completed*. |
| 3. Add a new assessment room with specified dimensions | Navigates to the room library and successfully creates a new assessment room by adding the name and dimensions. |
| 4. Explain the general purpose and initial instructions for the *R2Play* assessment to a new client | - Introduces rationale of *R2Play* (assessment to determine when youth are ready to return to play after concussion) - Explains purpose of *R2Play* (designed to feel like sport by combining physical and thinking skills) - Orients participant to system and general task (connect tablets in specific pattern based on numbers and letters displayed through several levels with added challenges) |
| *Administering assessment* | |
| 5. Begin a new assessment session with specified customization | - Initiates new assessment from home page   - *This step may be bypassed if assessment was already initiated during system connection (task #1)* - Selects the newly created athlete profile and room - Customizes trail difficulty using intermediate physical difficulty and advanced cognitive difficulty setting |
| 6. Administer baseline assessment of heart rate and symptoms | - Delivers instructions for PCSI and collects scores   - *Full PCSI does not need to be completed in training, just enough for clinician to demonstrate the task.* - Successfully connects the Polar monitor and collects resting heart rate by following the process outlined in the 4-step Polar HR connection guide handout |
| 7. Train a new client on a given level of the assessment | - Provides complete verbal instructions as shown in full script under “more details” on the level training page   - *Does not need to be verbatim, but should cover all key details (including repetition conditions)* - Plays and explains all trail sounds marked “new”   - *Playing other sounds is optional* - Offers participant the opportunity to view video demo and practice pattern using trace-it-out |
| 8. Document a clinical observation during an assessment | - Documents appropriate observation using the time-stamped notes function on right-hand side of screen   - *Can be done using pre-set options or custom write-in* |
| 9. Conduct a check-in assessment of RPE and symptoms | - Instructs participant how to provide RPE ratings using numeric, verbal, and visual anchors   - *Important to emphasize that RPE ratings are in relation to how participants felt during the level* - Instructs participant to provide symptom check-in response using verbal and visual cues   - *Important to emphasize that symptom check-in refers to changes in the symptoms previously rated on PCSI* |
| *Reviewing assessment results* | |
| 10. Report the peak and average heart rate that a client achieved in an assessment | - Reports correct peak and average heart rate from results summary screen |
| 11. Describe the change in a client’s symptoms during an assessment | - Reports correct change in participant’s full symptom score from pre- to post-assessment on PCSI tab   - *Participant may initially report the highest symptom check-in score from results summary screen; this is incorrect, and should be re-directed to PCSI* |
| 12. Explain a cost score from an assessment to a new client | - Identifies which levels/repetitions are being compared (i.e., Standard condition of Stroop level vs. standard condition of baseline number letter level   - *Important to briefly remind the youth participant what these tasks involve (e.g., connecting 1-A-2-B-3C trail on its own versus doing it with the colours)* - Describes numerical value of cost score (i.e., what was the percentage change?)   - *Can be done using any comparison metric, but is usually kept to default completion time metric* - Provides simple explanation of cost score result in easily understandable language (e.g., when you had to think more, your completion time was slower)   - *Important not to over-explain cost score results, as we do not know what these numbers mean in terms of return to play decisions and what is “normal”* |

PCSI, Post-Concussion Symptom Inventory; HR, Heart rate; RPE, Rating of perceived exertion.
