# Supplementary material for: Designing for implementation: development, delivery, and multi-method evaluation of a clinician training program for a novel technology-based concussion assessment tool
Source: Front Health Serv. 2026 Oct 1;6:1937910. doi: 10.3389/frhs.2026.1937910 (PMC13669162; doi:10.3389/frhs.2026.1937910)
Supplement: Supplementary file 4 [file Table3.docx]

**Supplementary Table 3.** Interview questions.

| Training | - What do you think about the training you were given to learn the *R2Play* assessment?   - How well do you think it prepared you to deliver the assessment? - What do you think about the content of the training session?   - Is there any other information you would have wanted to receive during training? - What do you think about the format of the training session?   - Were you able to learn the assessment effectively through the video modules and virtual training session?   - How helpful did you find the handout resources?   - Would you have preferred a different format of training? - How do you think we could improve the training? - Was there anything else beyond the formal training that helped you learn how to deliver the assessment? - If you were to implement this assessment in your practice, is there any further training or support that you think you would need? |
| --- | --- |
