# Supplementary material for: Designing for implementation: development, delivery, and multi-method evaluation of a clinician training program for a novel technology-based concussion assessment tool
Source: Front Health Serv. 2026 Oct 1;6:1937910. doi: 10.3389/frhs.2026.1937910 (PMC13669162; doi:10.3389/frhs.2026.1937910)
Supplement: Supplementary file 5 [file Table4.docx]

**Supplementary Table 4.** Qualitative analysis codes and quotes.

| **Domain and questions** | **Codes and frequencies** | **Sample quote** |
| --- | --- | --- |
| *Kirkpatrick Level 1: Reaction* | | |
| **Satisfaction**   - How favorable do learners find the training approach? - How satisfied are learners with the content and format of training? - What could be improved to make learners more satisfied? | General positive reactions (n=9) | “I think that you guys did a great job with doing the virtual training… I didn't have an issue with it being virtual. I thought it was fine.” – AT_1_, Site 1 |
|  | Sufficient training (n=15) | “Trained me enough, which doesn't sound positive, but I think that it was like a sufficient level of training to go into it.” – PT_2_, Site 2 |
|  | Online learning modules |  |
|  | Module resources are straightforward (n=5) | “Everything was really, really straightforward. The videos, even you put like the picture of the icons in the hands out. So, I did like everything.” – PTA_1_, Site 3 |
|  | Materials well-organized (n=1) | “But the material, they were well organized with the videos, with the numbers, with the, you know, everything was there.” – PTA_1_, Site 3 |
|  | Liked learning objectives and key take-aways (n=2) | “I appreciated the summaries afterwards, the preface of what we're going through today, or what this module is going through, and also the addition of written resources was fantastic.” – PT_3_, Site 2 |
|  | Written handout resources |  |
|  | Written handout resources helpful (n=8) | “The handouts were very helpful and very clear, very to the point, probably also one of the most useful tools that we got because we can just carry those around until we learn it. Kind of like any other assessment we use in clinic, we carry little cheat sheets until we figured it out. So the cheat sheets were very, very useful.” – PT_4_, Site 4 |
|  | Organize handouts into manual (n=2) | “I think it would be good at the end to have [the handout resources] all combined into one… cause when I did it then I had to go back to each module separately and print it off, it would just be easier to have the option of downloading like 1 PDF that is kind of the *R2Play* manual.” – PT_2_, Site 2 |
|  | Content gaps |  |
|  | Troubleshooting resources (n=4) | “Maybe a troubleshooting page? Like common places where clinicians can get held up like a FAQ of, like, make sure the Wi-Fi is OK, make sure the Bluetooth speaker is on… common questions or concerns or barriers to the setup process and implementation process.” – PT_3_, Site 2 |
|  | Interpreting results (n=4) | “The cost, like the analysis part was probably the one part that I probably could have read a little bit more of, we could have maybe discussed.” – PT_1_, Site 1 |
|  | Familiarization with the software interface (n=3) | “I think it was quite clear the only thing was just what happened if you press back or if you selected something different.” – PT_1_, Site 2 |
|  | Addressing unexpected issues (n=3) | “That human aspect… I remember the first participant, she had a question for me and I was like, I don't know how to answer this, it was just an extension of outside of what we learned on that online module and in person… and then I turned to you guys like I don't know kind of thing… it's just that like you just the things that you don't expect, the human aspect of like if they have a question of, I don't remember the question, but I have a question that is beyond kind of what we learned, I kind of I'm a little lost.” – PT_3_, Site 2 |
|  | Difficulty customizations (n=1) | “…looking at where those difficulty levels can be adjusted… I would just need to be like, wait, what does that actually look like again and see just a little bit more clearly the recap… I know it said it on there, but I just always clicked the one that I was supposed to do and kind of moved on.” – AT_2_, Site 1 |
| **Relevance**   - How relevant is training content to learners’ clinical experience? - How well does the training format meet the needs of learners? | Multimodal training satisfies learning preferences (n=4) | “I think that was also helpful to have the printout… I would draw things and write things. So again, you have your video, you stop it, you look at your handout, you're able to write things, but I think we all learn differently. So that was good.” – PT_2_, Site 4 |
|  | Training format aligns with healthcare education (n=1) | “In terms of medical or healthcare education overall, this is how we do it… we go through an online lecture portion, understand the basics and the foundation of what's going on and then we actually practise them one time in person, like a lab portion... So having a system do that I think is very familiar to me.” – PT_3_, Site 2 |
|  | Self-paced learning (n=3) | “It was really helpful to do the videos and we can go at our own pace. You can stop it, you can go back, it was really helpful.” – PT_2_, Site 4 |
|  | Segmented modules (n=3) | “I enjoyed the fact that they were split up, so it wasn't like a 2 hour just marathon video… clinicians just don't have X amount of hours in the day to sit through and fully attend to things, you get pulled aside and all that stuff and it's busy all the time.” – PT_3_, Site 2 |
|  | Time efficient (n=9) | “Didn't take too much of my time. I like that it gave me, like, the how long it would take so I could, you know, do this and then if I was busy, I would I would do the rest later.” – PT_2,_ Site 1 |
|  | Shorten videos (n=3) | “I think I would really cut it back to like two, three minutes… I'm older, but younger physios are all in the TikTok generation where they can only grab a certain amount of information at a time… seven minutes was reasonable.” PT_5_, Site 4 |
|  | Context on current practice (n=3) | “I think in a setting where not all the therapists have strong training in concussions... you have to have that background knowledge… unless they have an understanding of the basics, you can't just take that, open the box, give it to a patient and make a decision without understanding concussions.” – PT_5_, Site 4 |
| **Engagement**   - To what extent are learners actively involved in their learning experience? - Which training elements helped engage learners? - How interesting, compelling, or captivating do learners find the training? - What could help improve learners’ engagement in training? | Easy to understand (n=3) | “I thought it was easy to follow and understand.” –  OT_1_, Site 2 |
|  | Taking notes (n=1). | “So, with our handouts, I would write some physical notes here and there, as you were talking, so I was able to write things, so I think that was also helpful… you have your video, you stop it, you look at your handout, you're able to write things… So I was able to follow in the video, but also stop it in order for me to take a little few notes for me to make sure I understood.” – PT_2_, Site 4 |
|  | Re-reviewing material (n=14) | “I did refer back to it again a couple of times, like the modules. So I think it's good to still have that access to be like, okay, I need to go back and just refresh my memory or I need to go back and learn more about this concept.” – RN_1_, Site 2 |
|  | Using handouts during session (n=2) | “You had your like, how to set it up, like the one, two, three, four, five, six, seven, I think it was seven steps. And I just kind of would go through that. And then once you have it set up, I had the paper of what to do, but it kind of prompts you of what to do next, anyways, so I didn't really look at the paper. It was just the setting up that I always used the paper.” – PT_5_, Site 4 |
|  | Sharing experiences with colleagues (n=3) | “I guess as a group we did, we did exchange on our specific experiences, which did help. If you were the only person in that clinic to run this and you had no one to troubleshoot with, could be a little bit challenging.” – PT_1_, Site 4 |
| *Kirkpatrick Level 2: Learning* | | |
| **Knowledge**   - How well does training convey the information needed to deliver *R2Play*? - To what extent do learners obtain this knowledge? - Which aspects of training helped learners develop their knowledge of *R2Play*? - What could help learners better achieve this knowledge? | Modules provided foundational knowledge (n=14) | “Being able to do the modules first I think was a good intro. I think it was a great way to just learn about the concepts and learn about you know what is *R2Play* and what it does?” – RN_1_, Site 2 |
|  | Knowledge check questions reinforced learning (n=6) | “…the questions you guys asked underneath each of the videos were pretty beneficial and helped me remember what I had to look for and the key components of the modules for sure.” – AT_3_, Site 1 |
|  | Difficult to understand levels from module (n=2) | “I don't think anything was really missed, I think just the fatigue checks and maybe the Stroop trail, just the instructions felt a little unclear until I actually saw it. I think one of them even like did for the longer 1 and I still felt just a little unsure about it.” – AT_2_, Site 1 |
|  | Difficult to follow discrete videos (n=3) | “I did feel like the training modules kind of hopped around the assessment in in a certain way that for my brain was hard to follow.” – AT_1_, Site 1 |
|  | Seeing entire workflow (n=5) | “One thing I think would be beneficial is if there was a video of the entire process of how to set up, and taking like a participant through everything, I think that at the end of the thing would summarise and encapsulate and kind of bring everything together really well.” – PT_3_, Site 2 |
|  | Live training solidified understanding (n=12) | “The live training [did] help solidify what I need to know, and I think it was good.” – AT_3_, Site 1 |
| **Skills**   - How well does training teach the tasks that comprise delivery of *R2Play*? - To what extent do learners obtain these skills? - Which aspects of training helped learners develop these skills? - What could help learners better achieve these skills? | Learning by doing (n=3) | “It's great to see [the modules], but you really have to do it to learn it.” – PT_2_, Site 2 |
|  | Importance of hands-on practice (n=11) | “I definitely think an in-person demo is necessary. I think that was… what my team was saying after was the most useful in understanding how the tool is used and also trying it ourselves, I think made a big difference.” – PT_4_, Site 4 |
|  | Receiving feedback (n=1) | “I think it was nice to have the one-on-one and you guys being there kind of like evaluating me and giving your feedback on what I missed and how maybe I could change my explanation and whatnot… I thought it was nice to have that because it was a new system.” – PT_3_, Site 4 |
|  | Learning from mistakes (n=1) | “I knew what mistakes we had made the first day on the online in person training that when I did the actual [assessment session] it was yeah, it was pretty easy.” –  PT_1,_ Site 1 |
|  | Full assessment run-through (n=5). | “Maybe just running through an entire test with an actual athlete or an actual participant instead of just like with our research [staff]” – PT_4_, Site 4 |
|  | Shadowing experienced user (n=3) | “In a perfect world where we had to learn the system and be able to do it, I probably would have been like, hey [local research staff], can I just… shadow and watch you do a whole one through, and then gone and done the modules and then done a practice session, kind of the whole way through. I feel like watching a video could also work.” – AT_2_, Site 1 |
| Confidence   - How confident are learners during their *R2Play* assessment sessions? - To what extent do learners think they can successfully deliver *R2Play*? - What contributed to learners’ sense of confidence? - What could help make learners feel more confident? | Well prepared (n=6) | “I think [training] prepared me well… I've had two repetitions and I feel fairly comfortable with the programme already.” – PT_3_, Site 2 |
|  | Feeling confident (n=8) | “I felt confident.” – PT_2_, Site 4 |
|  | Adapting to new technology (n=3) | “It just takes time to be familiarize yourself with something new, like any new system that we use, you know, and then once you start using it, then it becomes easier and easier.” – PT_2_, Site 4 |
|  | Confidence increased with practice (n=5) | “Confidence went up quite a lot more for my second person.” – PT_2,_ Site 1 |
|  | Timing of training (n=8) | “I think I probably didn't remember things between when I did the training... I think it was the gap between when we were trained and when we did it was harder.” – PT_5_, Site 4 |
|  | Nervous about set-up (n=4) | “I was very nervous because of all of the, just the whole setting up and all that.” – PT_2_, Site 4 |
|  | Initially overwhelmed (n=5) | “The first time we were introduced to it, made it seem much more complicated than it was… there was a lot of tablets… like, oh my God, there's tablets. How do we set this up? What do we do now? We have to connect it to something… it just seemed like a lot of information at once and it was confusing…” – PT_5_, Site 4 |
|  | High-level overview video (n=4) | “…just having a basic, okay, this is what it looks like, this is the purpose of it. You know, this is what this looks like and this is what this looks like and this is whatever, before you even get into how to set it up.” –  PT_5_, Site 4 |
|  | Scoring comfort comes with experience (n=4) | “I think that again the cost… the analysis part was probably the one part that I probably could have read a little bit more… we could have maybe discussed. But I also think that you have to kind of do it a bit before you really get a feel for little scores and what's going on and you need to have a rationale for it.” – PT_1,_ Site 1 |
|  | Support from experienced user (n=19) | “I think talking through things with [local research staff], being able to ask questions was helpful… which I could see that being done in a clinic setting where someone the first time they're doing it has, you know, a clinician has run it before, just to like ask questions and show them.” – OT_1_, Site 2 |
