# Supplementary material for: Designing for implementation: development, delivery, and multi-method evaluation of a clinician training program for a novel technology-based concussion assessment tool
Source: Front Health Serv. 2026 Oct 1;6:1937910. doi: 10.3389/frhs.2026.1937910 (PMC13669162; doi:10.3389/frhs.2026.1937910)
Supplement: Supplementary file 6 [file Table5.docx]

**Supplementary Table 5.** Training module knowledge check questions.

**Module 1**

1) What are some of the limitations to the existing approach for return-to-play assessment after concussion that *R2Play* is intended to address? (Check all that apply).
   a) **Evaluating only one or two individual domains of function at a time.**
   b) **Assessments do not reflect the speed or complexity of sport.**
   c) **Difficulty identifying subtle changes in performance that only manifest during complex multi-task assessments.**
   d) **Limited evidence-based objective standards for decision-making.**
2) The purpose of *R2Play* is to…
   a) Help create a more sport-like assessment environment within a clinical setting.
   b) Simultaneously assess performance in physical, cognitive, perceptual, and psychosocial domains.
   c) Help inform return-to-play decision making among youth with concussion.
   d) **All of the above.**
3) *R2Play* is inspired by which traditional neuropsychological test?
   a) Continuous Performance Task.
   b) **Trail Making Test.**
   c) Mini-Mental State Test.
   d) Symbol Digit Modalities Test.
4) True or false: During *R2Play*, participants run between a series of tablets displaying numbers and letters to connect them in alphanumeric order (1-A-2-B-3-C…).
   a) **True.**
   b) False.

**Module 2**

1) The second repetition of each core *R2Play* assessment level is always:
   a) Standard condition.
   b) Auditory condition.
   c) Scramble condition.
   d) **Any of the above.**
2) Currently, all *R2Play* assessments use the intermediate setting for physical difficulty and advanced setting for cognitive difficult. This means that the trail sequence participants complete is:
   a) 1-A-2-B-3-C.
   b) 1-A-2-B-3-C-1-A-2-B-3-C.
   c) **1-A-2-B-3-C-4-D-5-E-6-F.**
   d) 1-A-2-B-3-C-4-D-5-E-6-F-7-G-8-H-9-I.
3) What are the 3 core *R2Play* assessment levels? (check all that apply).
   a) **Number-Letter trail.**
   b) **Exercise trail.**
   c) **Stroop trail.**
   d) Scramble trail.
   e) Auditory interference trail.
4) What happens during the scramble repetition condition of each *R2Play* level?
   a) The target trail pattern changes.
   b) **The numbers and letters unexpectedly change to a different tablet midway through the trail.**
   c) A distracting noise plays in the background throughout the trail.
   d) Some tablets appear in red and some appear in green.
5) Goals of the introductory phase of *R2Play* include (check all that apply):
   a) **Familiarize participant to the tablet system.**
   b) **Help participant learn the alphanumerical pattern.**
   c) **Gradually elevate heart rate.**
   d) **Warm-up relevant muscle groups.**

**Module 3**

1) Which of the following clinical measures is not monitored throughout *R2Play*?
   a) **Blood pressure.**
   b) Heart rate.
   c) Rating of perceived exertion (RPE).
   d) Concussion symptoms.
2) *R2Play* performance is measured based on:
   a) Speed (completion times in seconds/button).
   b) Accuracy (error counts).
   c) Multi-domain cost scores.
   d) **All of the above.**
3) Which of the following are true regarding *R2Play* cost scores? (check all that apply).
   a) **Cost scores reflect changes in performance between two levels or conditions with the addition of a new challenge.**
   b) Cost scores refer to changes in concussion-related symptoms from before to after completing *R2Play*.
   c) **Cost scores are calculated as the percentage increase in the reference performance** **metric between a baseline level or condition and a more challenging level or condition.**
   d) **Cost scores can be calculated using speed (completion time), accuracy (error), or heart rate.**
4) Using time as the comparison metric, an exertion cost score of 15% means…
   a) The participant was 15% faster during the standard condition of the Exercise Level compared to the standard condition of the Number-Letter Level.
   b) The participant was 15% slower during all repetitions of the Exercise Level compared to all repetitions of the Number-Letter level.
   c) **The participant was 15% slower during the standard condition of the Exercise Level compared to the standard condition of the Number-Letter Level.**
   d) The participant was 15% slower on the post-assessment fatigue check compared to the pre-assessment fatigue check.
5) Using errors as the comparison metric, a cognitive cost score of 25% means…
   a) The participant made 25% more errors during all scramble condition repetitions across each level compared to all standard condition repetitions across each level.
   b) **The participant made 25% more errors during the standard condition of the Stroop Level compared to the Standard condition of the Number-Letter Level.**
   c) The participant made 25% more errors during the Scramble condition of the Stroop Level compared to the Standard condition of the Stroop Level.
   d) The participant made 25% more errors during the Stroop condition of each level compared to the Standard condition of each level.
6) Using heart rate as the comparison metric, a scramble cost score of 5% means…
   a) The participant’s heart rate was 5% higher during all scramble condition repetitions compared to all auditory interference condition repetitions across each level.
   b) The participant’s heart rate was 5% higher during all standard condition repetitions compared to all scramble condition repetitions across each level.
   c) The participant’s heart rate was 5% higher during the scramble condition of the exercise level compared to the scramble condition of the Number-Letter level.
   d) **The participant’s heart rate was 5% higher during all scramble condition repetitions across each level compared to all standard condition repetitions across each level.**

**Module 4**

1) Common features across most of the *R2Play* screens include: (check all that apply)
   a) **Map icon.**
   b) **Up next indicator.**
   c) Save/export report.
   d) **Buttons for moving to next or previous screen.**
   e) **Home button.**
2) Customizing an *R2Play* assessment involves:
   a) Selecting client profile.
   b) Choosing assessment room.
   c) Customizing trail difficulty.
   d) **All of the above.**
3) At a minimum, which participant training method should be used for every level?
   a) **Verbal instructions.**
   b) Video demonstration.
   c) Trace-it-out.
   d) Listen to trail sounds.
4) Once an assessment level is started, you can still return to the level training page to review instructions with the participant.
   a) **True.**
   b) False.

**Module 5**

1) The *R2Play* system is comprised of: (check all that apply)
   a) **Clinician laptop.**
   b) **6 tablet buttons.**
   c) **Wi-Fi router.**
   d) **Heart rate monitor.**
   e) **Bluetooth speaker.**
   f) **Utility cart.**
2) Which network should the clinician laptop and tablet buttons be connected to?
   a) No network connections are needed.
   b) Local clinic Wi-Fi network.
   c) ***R2Play* network.**
   d) Either option.
3) When should the *R2Play* client be opened on the tablet buttons?
   a) As the tablets are placed in the stands and arranged out in the room (step 3).
   b) After the *R2Play* interface is opened on the clinician laptop (step 4).
   c) **After the system connection has been initiated on the clinician laptop (step 5).**
   d) It does not matter when the client is opened.
4) The *R2Play* client can be opened on the tablet buttons in any order.
   a) True.
   b) **False**.
5) When should the Polar heart rate connection be completed?
   a) During assessment set-up before the participant arrives.
   b) After the physical warm-up.
   c) **Once you arrive at the resting heart rate screen as part of pre-assessment measures collection.**
   d) During level training for the Number-Letter level.
